# Mask side-effects in long-term CPAP-patients impact adherence and sleepiness: the InterfaceVent real-life study

**DOI:** 10.1186/s12931-021-01618-x

**Published:** 2021-01-15

**Authors:** Marie-Caroline Rotty, Carey M. Suehs, Jean-Pierre Mallet, Christian Martinez, Jean-Christian Borel, Claudio Rabec, Fanny Bertelli, Arnaud Bourdin, Nicolas Molinari, Dany Jaffuel

**Affiliations:** 1 IMAG, CNRS, Montpellier University, Montpellier University Hospital, Montpellier, France; 2 Apard Groupe Adène, Montpellier, France; 3 Department of Respiratory Diseases, Montpellier University Hospital, Arnaud de Villeneuve Hospital, CHRU Montpellier, 371, Avenue Doyen Giraud, 34295 Montpellier Cedex 5, France; 4 Department of Medical Information, Montpellier University Hospital, Montpellier, France; 5 Inserm U1042, HP2 (Hypoxia PhysioPathology) LaboratoryCentre Hospitalier Universitaire Grenoble Alpes, Grenoble Alps University, Grenoble, France; 6 Pulmonary Department and Respiratory Critical Care Unit, University Hospital Dijon, Dijon, France; 7 PhyMedExp (INSERM U 1046, CNRS UMR9214), Montpellier University, Montpellier, France; 8 Pulmonary Disorders and Respiratory Sleep Disorders Unit, Polyclinic Saint-Privat, Boujan sur Libron, France

**Keywords:** Sleep apnea, Leaks, Side-effects, Telemedicine

## Abstract

**Background:**

For some patients, Continuous Positive Airway Pressure (CPAP) remains an uncomfortable therapy despite the constant development of technological innovations. To date, no real life study has investigated the relationship between mask related side-effects (MRSEs) and CPAP-non-adherence (defined as < 4 h/day) or residual-excessive-sleepiness (RES, Epworth-Sleepiness-Scale (ESS) score ≥ 11) in the long-term.

**Methods:**

The InterfaceVent-CPAP study is a prospective real-life cross-sectional study conducted in an apneic adult cohort undergoing at least 3 months of CPAP with unrestricted mask-access (34 different masks). MRSEs were evaluated using visual-analogue-scales, CPAP-data using CPAP-software, sleepiness using ESS.

**Results:**

1484 patients were included in the analysis (72.2% male, median age 67 years (IQ_25–75_: 60–74), initial Apnea–Hypopnea-Index (AHI) of 39 (31–56)/h, residual AHI_flow_ was 1.9 (0.9–4) events/h), CPAP-treatment lasted 4.4 (2.0–9.7) years, CPAP-usage was 6.8 (5.5–7.8) h/day, the prevalence of CPAP-non-adherence was 8.6%, and the prevalence of RES was 16.17%. Leak-related side-effects were the most prevalent side-effects (patient-reported leaks concerned 75.4% of responders and had no correlation with CPAP-reported-leaks). Multivariable logistic regression analyses evaluating explanatory-variable (demographic data, device/mask data and MRSEs) effects on variables-of-interest (CPAP-non-adherence and RES), indicated for patient-MRSEs significant associations between: (i) CPAP-non-adherence and dry-mouth (p = 0.004); (ii) RES and patient-reported leaks (p = 0.007), noisy mask (p < 0.001), dry nose (p < 0.001) and harness pain (p = 0.043).

**Conclusion:**

In long-term CPAP-treated patients, leak-related side-effects remain the most prevalent side-effects, but patient-reported leaks cannot be predicted by CPAP-reported-leaks. Patient-MRSEs can be independently associated with CPAP-non-adherence and RES, thus implying a complementary role for MRSE questionnaires alongside CPAP-device-reported-data for patient monitoring.

*Trial registration* InterfaceVent is registered with ClinicalTrials.gov (NCT03013283).

## Background

Sleep apnea syndrome (SAS) is a common sleep disorder with a prevalence ranging from 5.9% to 79.2% in European general populations over 35 years of age, depending on the clinical symptoms and apnea hypopnea scoring criteria used [1, 2]. To date, despite major advances in alternative therapies, Continuous Positive Airway Pressure (CPAP) remains the cornerstone of SAS treatment [3, 4]. Studies have shown that CPAP therapy can effectively reduce upper airway obstruction, with subsequent improvements in daytime sleepiness, sleep quality and quality-of-life, with all three of the latter being proportional to CPAP-usage [5, 6].

CPAP is an uncomfortable therapy for some patients. In large recent trials, CPAP-adherence (defined as a mean CPAP-usage for at least 4 h per day) can range from 53 to 92% at 6 months [7, 8]. In observational studies, the frequency of initial refusal of CPAP varies from 5 to 50% of patients [9]. Factors that influence adherence to CPAP include disease and patient characteristics, psychological and social factors, follow-up techniques (linked to healthcare professionals and healthcare facilities), governmental policies, technological device factors and, particularly, mask related side-effects (MRSEs) [10, 11].

Despite the constant development of technological innovations for improving mask comfort (e.g., mask shape, different breathing routes, materials, ergonomic straps, rotating tube intersections, and lighter masks), the impact of such innovations is uncertain. In an observational study published in 1995, 50% of the 193 patients complained of at least one MRSE [12]. Almost 20 years later, in the randomized controlled SAVE trial, 42% of patients complained of dry mouth [8]. In the latter study, the number of MRSEs (at 1 month) was an independent predictor of 12 month CPAP-adherence, thus confirming the relationship between the occurrence of short-term MRSEs and short-term CPAP-adherence. This relationship was confirmed in a 2311-patient real life cohort study, in addition to a significant relationship between CPAP-non-adherence and oronasal mask usage [13].

Similar data are lacking for long-term CPAP treated patients. Indeed, the 2019-published SAVE trial analysis [14] failed to demonstrate a relationship between MRSE-number and 24-month CPAP-adherence. However, it is difficult to extrapolate the results of randomized studies to real life because of differences between real life and randomized studies in SAS symptoms, SAS severity and the care protocol proposed (mask and CPAP choices/settings in particular). Unfortunately, other recent studies (randomized or real life) have not assessed the impact of MRSEs on long-term CPAP-adherence, nor on residual-excessive-sleepiness (RES, defined as an Epworth-Sleepiness-Scale (ESS) score ≥ 11) [15–17]. For real life, long-term CPAP-treated patients, the hypothesis that mask related side-effects (MRSEs) may affect CPAP-adherence or RES has never been investigated.

Therefore, the primary objective of the study reported herein is to describe MRSEs in a large population of SAS-patients undergoing long-term CPAP therapy under real life conditions (including an unrestricted access to masks and CPAP-devices available on the market). The secondary objectives are to evaluate MRSE effects on CPAP-adherence and RES.

## Methods

### Study design and study population

**Fig. 1:**
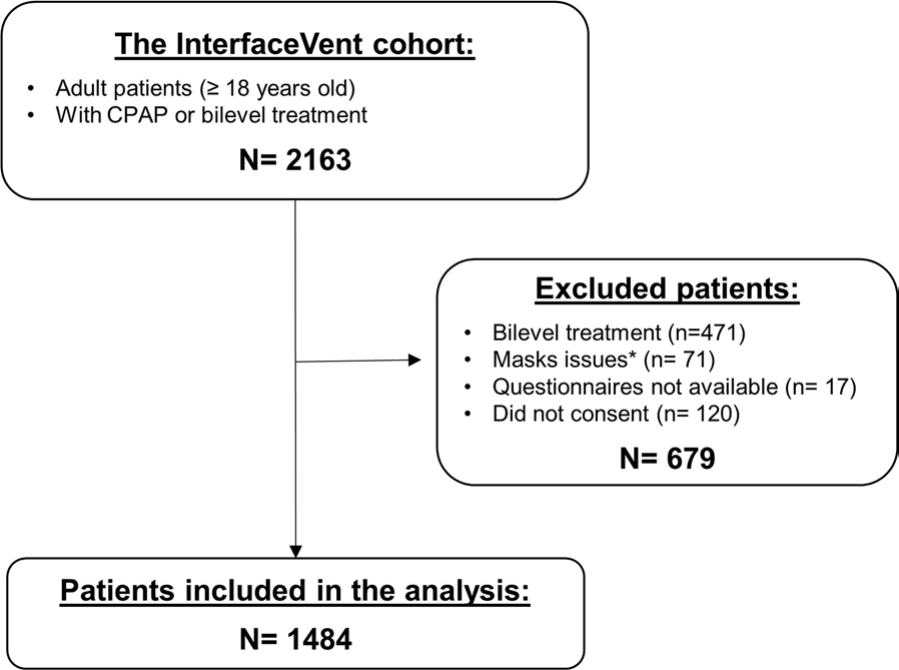
Study flow chart. CPAP: Continuous Positive Airway Pressure. *Multiple masks or mask-types not included (multiple mask-types for 66 patients, Liberty® mask for two patients, Oracle® oral mask for 3 patients)

The InterfaceVent study (ClinicalTrials.gov: NCT03013283) is a prospective real-life cross-sectional study conducted from February 7, 2017 to April 1, 2019 in adults undergoing at least 3 months of CPAP or non-invasive ventilation. We herein report results for SAS-patients treated exclusively by CPAP (see Fig. 1). SAS was defined according to the French Social Security (FSS) system criteria: (1) Apnea Hypopnea Index (AHI) ≥ 30/h (or AHI ≥ 15/h and more than 10/h respiratory-effort-related arousal), and (2) associated with sleepiness and at least three symptoms from among snoring, headaches, hypertension, reduced vigilance, libido disorders, nocturia). The respect of these criteria is a stipulation for reimbursement by the FSS.

Following an initial prescription by one of the 336 device-prescribing physicians in the *Occitanie* region of France, these patients were provided care by the Apard ADENE group, a non-profit home care provider. Patient inclusion was performed during a scheduled home visit by one of the 32 Apard technicians (visits were scheduled according to the visits required for the reimbursement of the CPAP treatment by the FSS single payer system). No CPAP-adherence threshold was required for reimbursement and patients with poor compliance were not systematically excluded (see Additional file 1 for exclusion criteria).

### Collected data

Clinical information collected for the analysis is exhaustively summarized in the Additional file 1.

For patients with a CPAP-duration > 6 months, data were collected only at the time of inclusion using CPAP-software and averaged over the last six months. For patients with 3–6 months CPAP, data were averaged for the last 3 months.

Side-effect visual analogue scales (VAS; see below), the Epworth-Sleepiness-Scale and the EQ-5D-3L questionnaire were administered during the scheduled visit by a technician employed by the home care provider. The technician did not help patients fill out the questionnaires. Residual Excessive Sleepiness (RES) was defined as an ESS score of ≥ 11.

An 11-point VAS (0 (-no reported side-effect) to 10 (very uncomfortable side-effect) was used to assess the following MRSEs: dry mouth, partner disturbance due to leaks, patient-reported leaks, noisy mask, heavy mask, painful mask, mask injury, painful harness, harness injury, redness of the eyes, itchy eyes, dry nose, stuffed nose, and runny nose. The presence/absence of aerophagia and nose bleeding were also reported using a binary question.

Device-reported leak variables, as stipulated by each manufacturer, were included in the analysis. These differ according to each manufacturer’s CPAP (see Additional file 2). For descriptive data, manufacturer-defined leak variables were used. For multivariable regression analyses, a pooled variable was used (see Additional files 3 and 4).

In order to compare masks on the basis of market availability, we divided them into two subgroups according to manufacturing date: those put on the market before 2013 (previous masks), versus those put on the market after 2013 (recent masks), see Additional file 5. This date was chosen based on the publication-year of the previous two large cohort studies evaluating the short-term impacts of MRSEs [13, 18].

### Statistical analyses

Continuous data were expressed as medians with their associated quartile ranges due to non-Gaussian distributions. Qualitative parameters were expressed as numbers and percentages.

Mask-types were compared using ANOVA or Kruskal–Wallis tests for quantitative data, and Chi-square or Fisher tests for qualitative ones. For significant global comparisons, pairwise comparisons with Holm corrections were performed. Multivariable logistic or linear regression analyses were used to study associations between different variables-of-interest (CPAP-non-adherence defined as a mean CPAP-usage for at least 4 h per day, Residual Excessive Sleepiness (RES, defined as an Epworth-Sleepiness-Scale (ESS) score ≥ 11), patient-reported leaks) versus explanatory-variables (demographic data, ESS score, EQ-5D-3L-questionnaires, device/mask data and MRSEs). Explanatory variables and multivariable regressions are exhaustively detailed in Additional files 6 and 7, respectively. To visualize correlations between MRSEs for a given mask type, a principal component analysis was performed for nasal, oronasal and nasal pillows masks [19, 20] (see Additional file 7). All statistical analyses were performed with SAS enterprise guide (V.7.1).

## Results

The flow chart of the study is depicted in Fig. 1. Briefly, a total of 1484 patients were included (72.2% male) in the analysis. The median age was 67 (IQ_25–75_: 60–74) years; the median body mass index was 31 (28–35) kg/m^2^; the median initial AHI was 39 (31–56)/h, and 11.9% were active smokers. The median duration of the CPAP treatment was 4.4 (2.0–9.7) years; the median CPAP-usage was 6.8 (5.5–7.8) h/day; the CPAP-adherence was lower than 4 h/day for 8.6% of the patients and the median residual AHI_flow_ was 1.9 (0.9–4) events/h. Overall, 28.4% of patients were treated with an oronasal mask (ONM), 54.4% with a nasal mask (NM), 17.2% with a nasal pillow mask (NPM), and 87.1% with auto-CPAP. The baseline characteristics of the population for each type of mask are summarized in Table 1. Additional file 8 shows the frequency distribution of the ESS scores in the whole population. The median ESS was 5 (3–9). The prevalence rate of RES (ESS score of ≥ 11) was 16.17% of the whole population.

**Table 1:** Population characteristics for each type of mask

	Whole Population (N = 1484)	Nasal (N = 807)	Oronasal (N = 422)	Nasal Pillows (N = 255)	p-value
Demographics					
Age (years)	67 [60; 74]	67 [61; 74]	68 [60; 74]	66 [59; 72]	0.089
Gender, female (%)	27.8	30.2^a^	21.3^b^	31.0^a^	0.002
BMI (kg/m^2^)	31 [28; 35]	31 [27; 35]	31 [28; 35]	30 [27; 35]	0.31
Diagnostic AHI (events/h)	39 [31; 56]	39 [31; 55]	39 [31; 58]	38 [31; 52]	0.86
Active smokers (%)	11.9	10.6	14.1	11.9	0.20
Beard (%)	17.0	17.3	17.4	15.1	0.22
Mustache (%)	10.4	8.5	13.4	10.8	
No mustache, no beard (%)	72.6	74.2	69.3	74.1	
Active workers (%)	20.6	19.3	20.5	24.8	0.18
Presence of partner (%)	72.2	71.2	73.1	73.9	0.64
Epworth Scale					
ESS (0–24 score)	5 [3; 9]	5 [3; 8]	6 [3; 9]	6 [3; 9]	0.16
RES (%)	16.2	15.1	17.1	18.0	0.46
EQ-5D-3L					
Problems with mobility (%)	24.4	22.5^a^	29.5^b^	21.9^a^	0.017
Problems with self-care (%)	6.0	5.5	7.0	6.1	0.59
Problems with usual activities (%)	19.7	19.5	20.6	18.4	0.78
Problems of pain/discomfort (%)	58.4	56.4	61.2	58.8	0.25
Problems of anxiety/depression (%)	39.4	34.7^a^	46.1^bc^	43.0^c^	< 0.001
EQ-5D-3L health VAS (0–100 score)	70 [50; 80]	70 [51; 80]^a^	68 [50; 80]^b^	70 [51; 81]^ac^	0.006
Device					
CPAP-usage (h/day)	6.8 [5.5; 7.8]	6.8 [5.6; 7.8]^a^	6.5 [5.3; 7.6]^bc^	6.6 [5.5; 7.6]^c^	0.022
Non-adherence (%)	8.6	6.3^a^	11.6^b^	11.0^b^	0.003
Current AHI_flow_ (events/h)	1.9 [0.9; 4.0]	1.7 [0.9; 3.7]^a^	2.7 [1.3; 5]^b^	1.6 [0.8; 3.0]^ac^	< 0.001
Treatment duration (yrs)	4.4 [2.0; 9.7]	4.4 [1.6; 10.1]	4.2 [2.2; 9.3]	5.1 [2.4; 9.7]	0.35
Mean Pressure (cmH_2_O)	8.2 [6.7; 10.0]	8.0 [6.4; 9.8]^a^	9.1 [7.6; 10.8]^b^	7.6 [6.5; 9]^ac^	< 0.001
90th/95th Pressure (cmH_2_O)	10.0 [8.1; 11.8]	9.9 [8.0; 11.4]^a^	11.0 [9.4; 12]^b^	9.6 [8; 11] ^ac^	< 0.001
Fixed pressure (%), n = 1483	12.9	13.0	12.8	12.9	0.99
Comfort mode (%)	15.8	16.2	14.7	16.1	0.77
Heated humidifier (%)	59.4	53.9^a^	68.3^b^	62.0^bc^	< 0.001
Heated breathing tube (%)	4.0	3.2	5.1	4.7	0.26
Mask					
Mask availability since 2013 (%)	41.2	29.8^a^	73.9^b^	39.8^c^	< 0.001
Unintentional leaks (l/min)	2.5 [0; 7.5]	2.5 [0; 8.4]^a^	1.2 [0; 6]^b^	1.5 [0; 7]^ab^	< 0.001
Unintentional large Leaks (%)	0.1 [0; 0.9]	0.1 [0; 0.6]	0.1 [0; 2.5]	0.1 [0; 0.7]	0.08
Global leaks (l/min)	33 [27; 41]	31.5; 26; 37]^a^	37 (32; 49]^b^	32 [26; 41]^ab^	0.014
Global large leaks (%)	0.9 [0.1; 5.1]	0.7 [0.1; 3.1]	2.7 [0.2; 6.7]	0.9 [0.2; 5]	0.11
Chin strap (%)	0.47	0.50	0.47	0.39	0.98

AHI: Apnea–Hypopnea Index; AHI_flow_: AHI reported by device; BMI: Body Mass Index; CPAP: Continuous Positive Airway Pressure; ESS: Epworth Sleepiness Scale; N = number of patients responding; Non adherence: CPAP-usage under 4 h per day; RES: Residual Excessive Sleepiness (ESS score > 10); VAS: Visual Analogue Scale. Leaks were obtained using CPAP built-in software (See Additional file 2: Table S1 for details). Data are reported as medians and quartiles or numbers and percentages of total as appropriate

Labels ^a^, ^b^, ^c^: within a given line, mask-type subgroups with different letters are significantly different (p < 0.05) according to post-hoc pairwise comparisons after Holm corrections. As an example, for the gender variable, there is a significant difference (p = 0.002) between mask-types. Post-hoc pairwise comparisons indicate that nasal-mask type is significantly different with oronasal-mask type (labels ^a^ and ^b^ are different), nasal pillows-mask type is also significantly different with oronasal-mask type (labels ^a^ and ^b^ are different) but there is no significant different between nasal and nasal pillows-types (same label ^a^)

Additional file 5 summarizes the number of patients by brand, model and type of mask and Additional file 9 summarizes the brand, series and mode of CPAP devices. The prevalence of aerophagia and nose bleeding in our whole population were respectively 8.28% and 4.16%, with no significant differences between mask-types (respectively p = 0.47 and p = 0.75).

### Prevalence of mask related side-effects

**Fig. 2:**
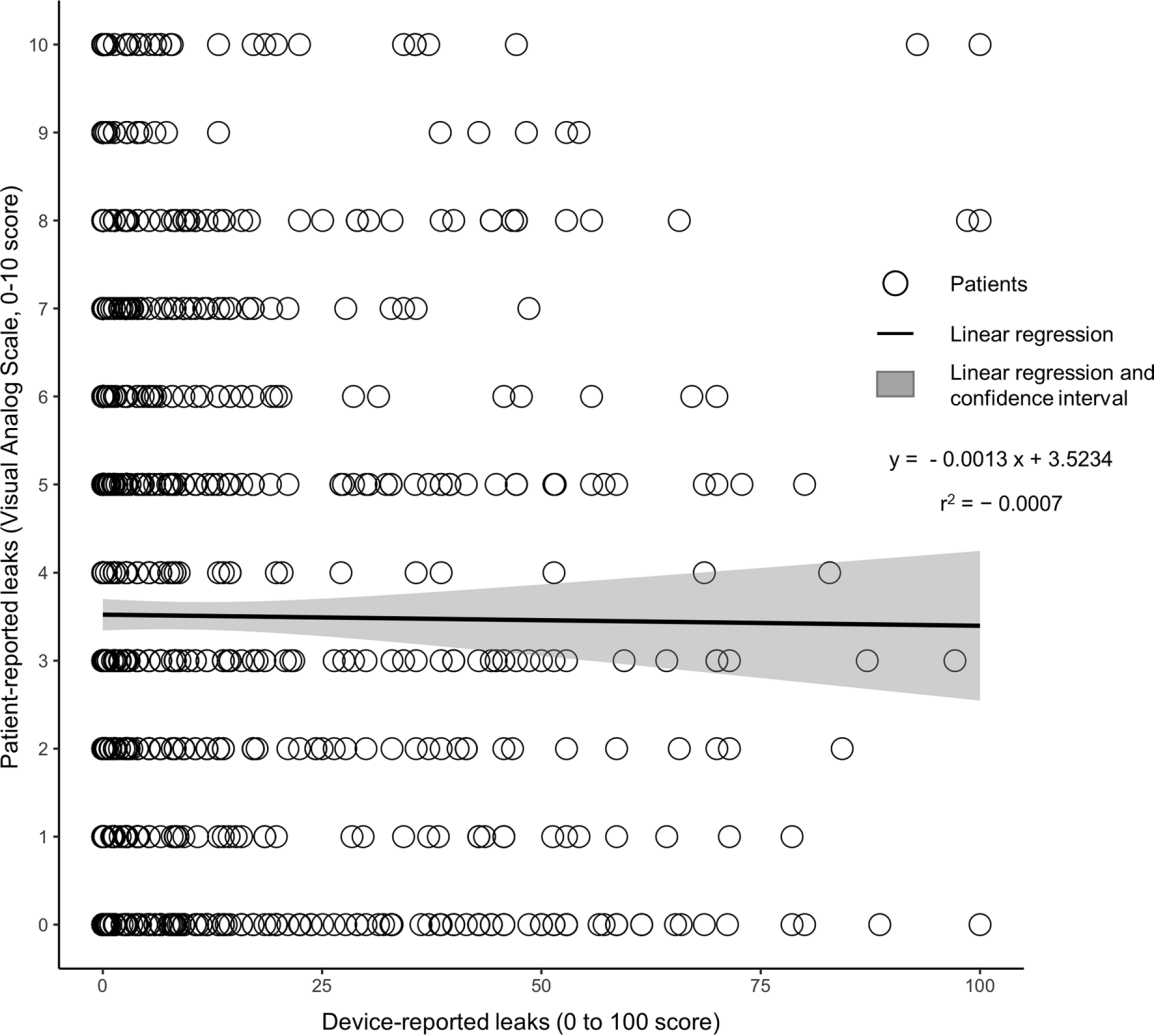
Relationship between patient-reported leaks and device-reported leaks: individual data and linear regression

MRSE frequencies (VAS score ≥ 1 and VAS score ≥ 5) are summarized in Table 2. Leak-related side-effects are the most prevalent type of side-effect. In particular, patient-reported leaks concerned 75.4% of the respondents. There is no association between patient-reported and device-reported leaks (the Pearson correlation coefficient *r* is only 0.007 (p = 0.78), the regression coefficient is only -0.0013 (p = 0.79), see Fig. 2 and Additional file 10). Multivariable linear regression (Additional file 11) indicated that dry mouth, partner-disturbing leaks, noisy mask and stuffed nose were all significantly associated with patient-reported leaks regardless of the model considered (contrary to CPAP device-reported variables).

**Table 2:** Prevalence of mask related side-effects

Mask related side-effects	% of patients reporting VAS ≥ 1 (% of population responding positively to the question)	% of patients reporting VAS ≥ 5 (% of population responding positively to the question)
Patient-reported leaks (%)	75.4	39.5
Dry mouth (%)	70.6	42.5
Partner-disturbing leaks (%)	69.4	39.0
Noisy mask (%)	57.5	22.6
Dry nose (%)	54.4	25.6
Stuffed nose (%)	41.7	17.6
Red eyes (%)	36.2	14.7
Itchy eyes (%)	36.0	13.1
Runny nose (%)	36.0	14.0
Heavy mask (%)	36.0	5.81
Mask pain (%)	31.9	7.43
Mask injury (%)	27.6	6.48
Harness pain (%)	27.3	4.72
Harness injury (%)	22.4	2.54

VAS: Visual Analogue Scale

**Fig. 3:**
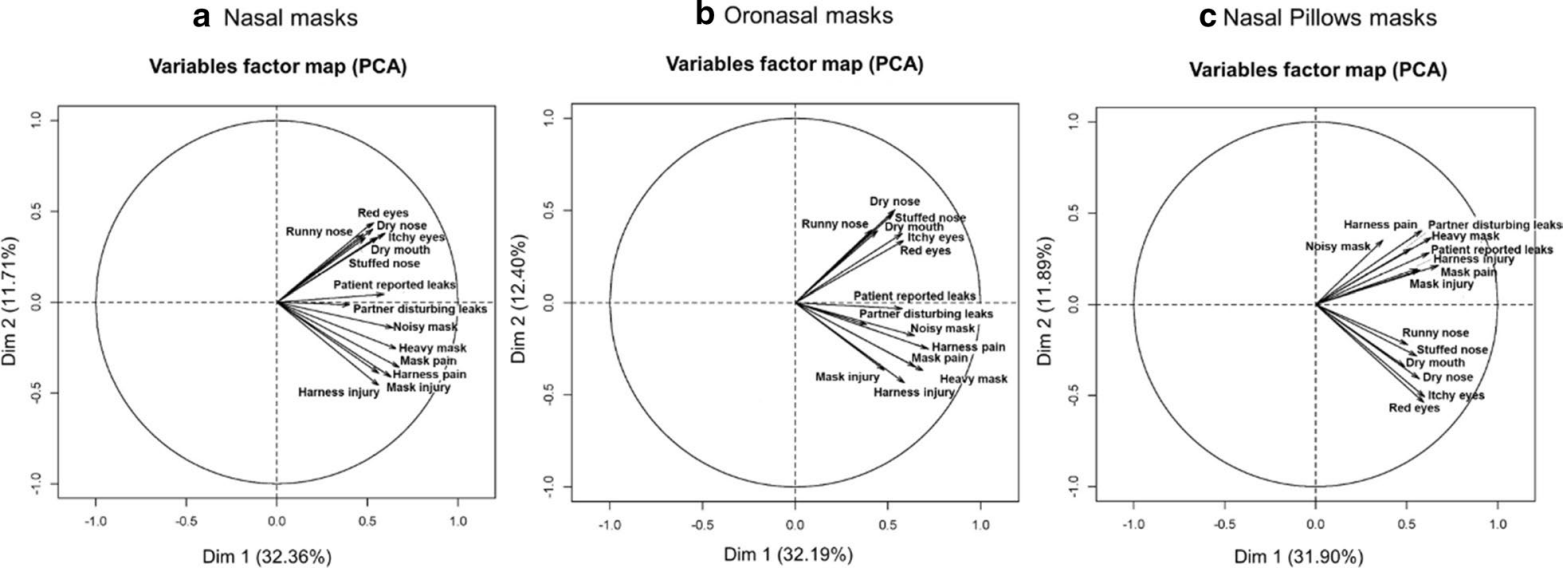
Principal Component Analysis (PCA) of the mask related side-effects for **a** nasal, **b** oronasal, **c** nasal pillows masks. Note that PCA is a procedure that transforms possibly correlated variables into a smaller number of uncorrelated variables called principal components. In this process, linear relationships among variables are found (components), with each component being uncorrelated with the others (orthogonal) in directions defined by an eigenvector. The first principal component accounts for as much of the variability in the data as possible, and each succeeding component accounts for as much of the remaining variability as possible (with the restriction of being orthogonal to/independent of the preceding component). Within this framework, the reader should understand that two arrows pointing in the same direction and close to each other are positively correlated, two arrows pointing in opposite directions are negatively correlated, and arrows at right angles are independent. Here, the three plots present a similar explained total variance for PC1 of 31.90% to 32.36%, PC2 of only 11.7% to 12.4% and only minor differences in positioning of the vectors for each MRSE on the 3 plots. In consequence, PCA analyses suggest few differences for MRSE associations between mask types. Independently of the mask-type, two groups of eigenvectors can be described. The first group is composed of red eyes, itchy eyes, dry nose, runny nose, stuffed nose and dry mouth eigenvectors (PCA group-1). The second group is composed of mask pain, harness pain, mask injury, harness injury, heavy mask, patient reported leaks, partner disturbing leaks and noisy mask eigenvectors (PCA group-2). These two groups are independent between them whereas their constitutive MRSE variables are associated. For nasal pillow masks, the group-2 composition is similar but with an increased association between constitutive MRSE variables

**Fig. 4:**
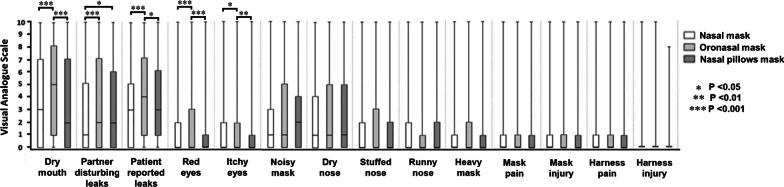
Visual Analogue Scales for mask related side-effect scores (0–10 score) according to mask type

In the same patient, MRSEs can coexist. Additional file 12 summarizes the percentage of patients with 0 to 14 associated MRSEs. Principal Component Analysis (PCA) results (see Fig. 3, panel a) for NMs, panel b) for ONMs and panel c) for NPMs) suggest MRSE correlations that are homogenous across mask-types. However, Fig. 4 indicates additional mask differences in terms of specific VAS scores. For example, when comparing ONM to NM, higher MRSE VAS scores were found for patient-reported leaks (p < 0.001), partner-disturbing leaks (p < 0.001), dry mouth (p < 0.001), red eyes (p = 0.005) and itchy eyes (p = 0.02). Similarly, when comparing ONM to NPM, higher MRSE VAS scores were found for patient-reported leaks (p = 0.04), dry mouth (p < 0.001), red eyes (p < 0.001), and itchy eyes (p = 0.001). Finally, when comparing NPM to NM, higher MRSE VAS scores were found for partner-disturbing leaks (p = 0.013) only. In addition, the PCA suggests that the group of MRSE associated with mask complaints (PCA group-2) may be associated with the presence of a bed partner.

Additional file 13 depicts VAS for MRSE scores according to a bed partner presence or not. Noisy mask (p < 0.001) and mask pain (p < 0.05) are significantly associated with bed partner presence.

The MRSEs according to mask type and market availability are depicted in Additional file 14. Briefly, NMs available after 2013 provoke more red-eye symptoms than older masks. Recent ONMs are significantly more associated with partner-disturbing leaks and dry nose than older ones. Recent NPMs are also more associated with red eyes and itchy eyes than previous ones.

### Mask related side-effects, CPAP-non-adherence and residual-excessive-sleepiness

The quasi-totality of patients (1467/1478; 99.3%) position masks without assistance (97.1% (1424/1466) easy to pose), 94.9% (1385/1460) were satisfied with their mask, 90.4 (1311/1451) of the patients considered masks as comfortable.

Table 4 summarizes the multivariable logistic regression analyses evaluating the impact of explanatory variables on CPAP-non-adherence and RES (see Table 3 for univariate analyses). In both models explaining CPAP-non-adherence, the latter was independently associated with lower age, lower BMI, lower p90/95^th^ pressure, availability of the mask since 2013, higher dry mouth and lower partner-disturbing leaks. Regardless of the model specification used, RES (Table 4) was independently associated with stronger anxiety/depression (estimated via the EQ-5D-3L anxiety/depression domain), lower quality-of-life (EQ-5D-3L-health VAS score), and higher VAS scores for noisy mask, patient-reported leaks, dry nose and harness pain.

**Table 3:** Univariate logistic analyses

	CPAP-non-adherence (< 4 h/day)		Residual Excessive Sleepiness (ESS ≥ 11)	
	Crude OR [95% CI]	P value	Crude OR [95% CI]	P value
Demographics				
Age (years)	*0.98 [0.97; 1.00]*	*0.046*	0.*99 [0.97; 0.99]*	*0.02*
Gender, female	*0.55 [0.34; 0.80]*	*0.002*	1.10 [0.80; 1.50]	0.55
BMI (kg/m^2^)	*0.95 [0.92; 0.99]*	*0.012*	0.99 [0.96; 1.01]	0.27
Diagnostic AHI (events/h)	1.00 [1.00; 1.00]	0.25	0.99 [0.99; 1.00]	0.31
Active smokers	1.40 [0.84; 2.36]	0.20	1.26 [0.84; 1.89]	0.27
Beard	0.80 [0.40; 1.60]	0.31	1.34 [0.88; 2.04]	0.10
Mustache	1.35 [0.66; 2.74]	0.27	0.80 [0.44; 1.46]	0.23
Active workers	*1.50 [0.98; 2.29]*	*0.059*	*1.36 [0.98; 1.89]*	*0.07*
Presence of bed partner	*0.51 [0.35; 0.74]*	*< 0.001*	0.93 [0.69; 1.27]	0.65
Epworth Scale				
ESS (0–24 scores)	*1.00 [0.99; 1.00]*	*0.005*	NA	NA
EQ-5D-3L				
Problems with mobility	1.18 [0.78; 1.78]	0.44	*1.55 [1.14; 2.11]*	*0.005*
Problems with self-care	1.22 [0.60; 2.51]	0.58	*1.50 [0.89; 2.56]*	*0.13*
Problems with usual activities	1.21 [0.78; 1.89]	0.39	*2.06 [1.50; 2.83]*	*< 0.001*
Problems of pain/discomfort	1.10 [0.76; 1.60]	0.62	*1.79 [1.33; 2.42]*	*0.001*
Problems of anxiety/depression	1.23 [0.84; 1.78]	0.29	*2.42 [1.82; 3.21]*	*< 0.001*
EQ-5D-3L health VAS (0–100 score)	*1.01 [0.97; 1.01]*	*0.071*	*0.99 [0.99; 1.00]*	*< 0.001*
Device				
CPAP-usage (h/day)	NA	NA	*0.99 [0.99; 1.00]*	*0.001*
Current AHI_flow_ (events/h)	*1.04 [0.99; 1.09]*	*0.054*	*0.99 [0.95; 1.04]*	0.39
Treatment duration (years)	*0.93 [0.89; 0.97]*	*< 0.001*	*1.00 [0.98; 1.03]*	0.93
Fixed pressure	*0.49 [0.24; 0.98]*	*0.043*	*0.67 [0.43; 1.07]*	*0.10*
Mean pressure (cmH_2_O)	*0.92 [0.84; 1.00]*	*0.058*	*0.98 [0.92; 1.05]*	0.68
90th/95th pressure (cmH_2_O)	*0.93 [0.85; 1.01]*	*0.072*	*1.02 [0.96; 1.09]*	0.43
Comfort mode	1.33 [0.84; 2.11]	0.22	0.90 [0.61; 1.32]	0.58
Heated humidifier	*1.45 [0.98; 2.12]*	*0.061*	1.12 [0.84; 1.49]	0.43
Heated breathing tube	1.71 [0.79; 3.68]	0.17	1.06 [0.53; 2.12]	0.87
Mask				
Nasal mask	*Ref*	*0.003*	Ref	0.46
Oronasal mask	*1.95 [1.29; 2.94]*	*0.0625*	1.16 [0.84; 1.59]	0.81
Nasal pillows mask	1.83 [1.13; 2.97]	0.23	1.23 [0.85; 1.79]	0.44
Availability of the mask since 2013 (%)	*2.49 [1.72; 3.61]*	*< 0.001*	1.21 0.92; 1.60]	0.17
Device reported leaks (0–100 score)	1.0 [0.99; 1.02]	0.23	*1.00 [0.99; 1.00]*	0.67
Device reported leaks (median of the 95th percentile of unintentional leaks (l/min))	1.01 [0.99; 1.02]	0.34	0.99 [0.98; 1.01]	0.28
Chin strap	1.00 [1.00;1.00]	0.98	*3.92 [0.87; 17.6]*	*0.07*
Side effects				
Dry mouth (0–10 VAS score)	*1.05 [1.00; 1.11]*	*0.044*	*1.14 [1.09; 1.18]*	*< 0.001*
Partner disturbing leaks (0–10 VAS score)	*0.89 [0.84; 0.95]*	*< 0.001*	*1.08 [1.05; 1.13]*	*< 0.001*
Patient reported leaks (0–10 VAS score)	1.00 [0.94; 1.06]	0.96	*1.15 [1.10; 1.21]*	*< 0.001*
Red eyes (0–10 VAS score)	1.01 [0.94; 1.08]	0.86	*1.10 [1.05; 1.15]*	*< 0.001*
Itchy eyes (0–10 VAS score)	0.99 [0.92; 1.07]	0.90	*1.11 [1.05; 1.16]*	*< 0.001*
Noisy mask (0–10 VAS score)	0.99 [0.93; 1.07]	0.90	*1.16 [1.10; 1.22]*	*< 0.001*
Dry nose (0–10 VAS score)	*1.04 [0.99; 1.10]*	*0.14*	*1.15 [1.10; 1.20]*	*< 0.001*
Stuffed nose (0–10 VAS score)	1.01 [0.94; 1.08]	0.81	*1.13 [1.09; 1.19]*	*< 0.001*
Runny nose (0–10 VAS score)	0.97 [0.90; 1.05]	0.44	*1.06 [1.01; 1.12]*	*0.013*
Heavy mask (0–10 VAS score)	0.92 [0.81; 1.04]	0.18	*1.20 [1.12; 1.28]*	*< 0.001*
Mask pain (0–10 VAS score)	1.05 [0.96; 1.15]	0.25	*1.10 [1.03; 1.20]*	*0.003*
Mask injury (0–10 VAS score)	1.01 [0.91; 1.11]	0.90	*1.11 [1.04; 1.18]*	*0.002*
Harness pain (0–10 VAS score)	1.07 [0.96; 1.18]	0.21	*1.11 [1.03; 1.20]*	*0.006*
Harness injury (0–10 VAS score)	0.93 [0.79; 1.09]	0.36	*1.12 [1.03; 1.21]*	*0.01*
Nose bleeding	0.53 [0.17; 1.73]	0.30	1.16 [0.60; 2.27]	0.66
Aerophagia	1.45 [0.80; 2.61]	0.22	*1.55 [0.99; 2.44]*	*0.056*
Number* [0/14]	1.01 [0.96; 1.05]	0.84	*1.12 [1.08; 1.16]*	< 0.001

AHI: Apnea–Hypopnea Index, BMI: body mass index, CPAP: continuous positive airway pressure, ESS: Epworth Sleepiness Scale, OR: odds ratio; NA: not applicable, VAS: Visual Analogue Scale

Italics variables are included in multivariable analyses

* Number of side effects; dichotomous data created when the VAS scale for the side effect was above or equal to 1. This variable was not included in the multivariable analyses because of its collinearity with MRSE-variables

**Table 4:** Multivariable logistic regressions for CPAP-non-adherence and Residual Excessive Sleepiness as variables-of-interest, summary of significant explanatory-variables including side-effects

	CPAP-non-adherence (< 4 h/day)				Residual Excessive Sleepiness (ESS ≥ 11)					
	Model 1		Model 2		Model 1		Model 2		Model 3	
	OR [95% CI]	p-value	OR [95% CI]	p-value	OR [95% CI]	p-value	OR [95% CI]	p-value	OR [95% CI]	p-value
Age (years)	0.98 [0.96; 0.99]	0.008	0.98 [0.96; 0.99]	0.008						
BMI (kg/m^2^)	0.95 [0.91; 0.99]	0.008	0.95 [0.92; 0.99]	0.008						
Problems of anxiety/depression					1.87 [1.25; 2.81]	0.002	1.92 [1.27; 2.88]	0.002	1.92 [1.27; 2.88]	0.002
EQ-5D-3L health VAS (0–100 score)					0.98 [0.97; 0.99]	< 0.001	0.98 [0.97; 0.99]	< 0.001	0.98 [0.97; 0.99]	< 0.001
90th/95th Pressure (cmH_2_O)	0.90 [0.81; 0.99]	0.028	0.90 [0.81; 0.99]	0.025						
Oronasal Mask			1.33 [0.79; 2.23]	0.80			0.74 [0.48; 1.16]	0.269	0.74 [0.48; 1.16]	0.269
Nasal Pillows Mask			1.55 [0.88; 2.73]	0.274			0.90 [0.53; 1.54]	0.846	0.91 [0.53; 1.54]	0.847
Chinstrap					7.22 [1.08; 48.4]	0.0417	7.27 [1.09; 48.55]	0.0406	7.27 [1.09; 48.5]	0.040
Mask availability since 2013	2.42 [1.56; 3.74]	< 0.001	2.28 [1.45; 3.59]	< 0.001						
Dry mouth (0–10 VAS score)	1.10 [1.03; 1.17]	0.003	1.10 [1.03; 1.17]	0.004						
Patient-reported leaks (0–10 VAS score)					1.10 [1.03; 1.19]	0.009	1.11 [1.03; 1.19]	0.007	1.11 [1.03; 1.20]	0.007
Partner-disturbing leaks (0–10 VAS score)	0.86 [0.82; 0.93]	< 0.001	0.86 [0.80; 0.92]	< 0.001						
Noisy mask (0–10 VAS score)					1.16 [1.07; 1.25]	< 0.001	1.16 [1.07; 1.26]	< 0.001	1.16 [1.07; 1.26]	< 0.001
Dry nose (0–10 VAS score)					1.13 [1.06; 1.20]	< 0.001	1.13 [1.06; 1.20]	< 0.001	1.13 [1.06; 1.20]	< 0.001
Harness pain (0–10 VAS score)					0.89 [0.79; 1.00]	0.044	0.89 [0.79; 1.00]	0.002	0.89 [0.79; 1.00]	0.043

AHI: Apnea–Hypopnea Index; AHI_flow_: AHI reported by device; BMI: body mass index; CPAP: Continuous Positive Airway Pressure; ESS: Epworth Sleepiness Scale; NA: not applicable; OR: odds ratio; VAS: Visual Analogue Scale. Note that for model 1, explanatory-variables (exhaustively listed in Table 3) with a p-value < 0.15 at the univariate level were fed into multivariable analyses using stepwise selection. A backward elimination was then applied and only explanatory-variables with a p value < 0.05 at the multivariable level remain in the definitive models. For model 2, the same proceeding as model 1 was applied and the “mask-type” explanatory variable was forced (with “nasal” as the reference mask type). For model 3, the same procedure as model 2 was applied, but the interaction terms between CPAP-usage and significant MRSEs from model 2 were added and a backward elimination applied excepted for the “mask-type” variable which remains forced. Note that no interaction term between CPAP-usage and MRSE variables remained significant in the final model 3

## Discussion

To our knowledge, this study is the first to report the prevalence of several MRSEs and their impact on CPAP-non-adherence and RES in a large cohort of long-term CPAP-treated patients. Patients were treated under real life conditions with an unrestricted access to masks and market-available CPAP devices. The main results reported here suggest that: (1) the most frequent side-effects in long-term CPAP-treated patients are patient-reported leaks, which are not predicted by CPAP-device-reported data; (2) in long-term CPAP-treated patients, MRSEs are independently negatively associated with CPAP-adherence and positively associated with sleepiness, contrary to certain device-reported variables (CPAP-AHI_flow_ and CPAP-reported leaks), which are not.

Prior to the availability of humidification systems, airway dryness was the most frequently reported side-effect (> 50% of CPAP-treated patients) [21]. In the 2000s, skin abrasions and mask leaks were considered as the most prevalent [22]. In 2013, two large cohorts reported MRSEs in short-term CPAP-treated patients [13, 18]. Additional file 15 summarizes the main data provided by these two studies; the most frequent side-effects were leak-related, as also found in the current study. In addition, we confirm that patient-reported leaks are not predicted by CPAP-reported leaks [18]. CPAP devices only record leak flows, whereas the patient's perception of a leak is a complex phenomenon involving not only the strength of the leak-flow itself but also how it feels on his/her skin/eyes (if the mask is not properly adjusted), and associated noises. In this regard, it is important to note that beards and moustaches were not associated with patient-reported leaks (as previously reported by Bachour et al. [18]).

Comparing the prevalence of MRSEs between studies is not simple. There are differences in the type of side-effects collected, as well as differences in the variables used (yes/no binary questionnaires, VAS and Likert scales). In addition, how patients perceive an MRSE is a complex phenomenon. Indeed, in the same study, the prevalence of MRSEs was reported to be different between a Caucasian population and an Asian population without a clear explanation why (cultural differences and/or reporting biases was preferentially suggested, rather than anatomical and/or physiological variations) [8]. Moreover, differences exist not only between mask-types, but also between brands or even series [18, 23]. There is a need for standardized questionnaires with a VAS or Likert scale rather than binary questions. Binary questions tend to censor positive responses from patients with less-severe complaints and thereby reduce the accuracy of statistical analyses. These limits were underlined in the 2019-American Academy of Sleep Medicine (2019-AASM) systematic review of CPAP-treatment for SAS patients [4].

Among the many factors that influence adherence of CPAP-treated patients, a good mask-type choice is of critical importance [24]. Two reviews and meta-analyses have reported a lower CPAP-adherence with ONMs compared with NMs, but they underlined that this difference was only observed in cohorts but not in randomized studies [4, 25]. Considering that MRSEs are different between mask-types [4], one hypothesis that likely explains differences between mask-types on CPAP-adherence might be that such differences are more the result of MRSEs than the mask-type per se. In this regard, at the multivariable level of analysis (model 1), we report that dry-mouth is associated with poor CPAP-non-adherence (< 4 h/day), whereas the mask-type was not. Of course, it was impossible for us to rule out the possibility that a certain collinearity between MRSEs and mask type limits the statistical analysis. Previous studies reporting on how MRSEs impact CPAP-adherence concern only NM [26] or do not include mask-type as a variable of study [8, 27]. Only Borel et al. included both MRSEs and mask-type in a multivariable analysis and found that ONM versus NM effects associated with CPAP-adherence, while dry mouth and nasal congestion became insignificant at the multivariable level [13]. The observed discrepancies between the present study and that by Borel et al. [13] can be explained by differences in study design, such as long versus short-term timeframes (fewer stable patients and fewer attempts at using different masks), or by the use of quantitative VAS scores versus a binary questionnaire that likely censors information.

We reported that the patients with an ONM or a NPM have a higher CPAP non-adherence than the patients with a NM. Similar results were observed in both a cohort study by Borel et al. [13] and in a 4-week randomized cross-over study by Goh et al. [28]. On the contrary, Rowland et al. found no significant difference between the mask-types on CPAP non-adherence when comparing NM and ONM in a 4-week randomized cross over trial [29]. To explain certain similarities and discrepancies between these studies and ours, it is important to keep in mind that: (i) in accordance with the 2010 French national recommendations, the mask-type national policy was to use a NM as the first intention mask in newly CPAP-treated patients [30]; (ii) in our study, the patients had access to 34 different masks and were treated on a long-term basis (median CPAP-treatment duration of 4.4 [IQ_25;75_: 2.0; 9.7] years, with an unrestricted mask-type use and potentially several mask-type sequences before inclusion). So we cannot rule out that our reported CPAP non-adherence might be impacted by this initial choice of a NM and/or the different mask-type sequences. For most patients, NPM and ONM choices were probably an alternative/catch-up choice because of a NM problem, rather than a first choice.

We also included CPAP-reported variables in our multivariable regression models. Our study confirms that higher levels of CPAP-pressure are predictive of higher compliance [13, 14, 31, 32], but neither AHI_flow_ nor device-reported leaks were significantly associated with CPAP-non-adherence in these long-term CPAP-treated patients. However, dry mouth is. Furthermore, we report ESS scores to be significantly associated with MRSEs whereas AHI_flow_ and device-reported leaks are not. Altogether, these results suggest that MRSE questionnaires should be included with CPAP-reported data during patient follow-up and particularly in long-term tele-monitoring programs [33].

We reported that the CPAP-mode (fixed versus auto-adjusting pressure) has no impact on CPAP-non adherence nor on RES. These data are in accordance not only with the results of a recent meta-analysis of two randomized clinical trials on CPAP-non adherence, but also with 19 studies on ESS [4].

### Study limitations

One part of our results can be explained by an inherent bias in the real-life design of our study. We not only report that “mask availability since 2013” is associated with CPAP-non-adherence (< 4 h/day), but also that MRSEs are more prevalent with recent. This finding could be partly explained by the fact that patients with previous/prominent MRSEs may have been more likely to have changed/upgraded masks and thus have a newer one at the time of the study. Another explanation could be that very long-term treated patients were historically treated with “older-masks”. During planned technician visits, these patients may also have tried “recent-masks”, whereas for newer patients, only the more “recent-masks” are proposed in order to limit the risk of rapid obsolescence. Bachour et al. reported that 20% of patients were unsatisfied with their newer mask when a systematic switch was necessary because of obsolescence [23]. Thus, only mask-incident patients and a randomized trial design would resolve questions concerning the benefits of old-versus-new masks. It is also important to underline that newer mask-types such as nasal cradles or minimal contact nasal/oronasal mask-types were very rarely or not used in our study, so our findings do not apply to these newer mask-types.

That 95% and 90% of patients were satisfied with their mask or considered it comfortable (respectively), whereas > 75% reported ≥ 1 MRSE, presents a paradox. We believe this is a consequence of (i) the 11-point MRSE VAS used in our study (for most patients, a MRSE exists but is not considered “uncomfortable or very uncomfortable”); (ii) patients are long-term treated (> 75% of patients are treated ≥ 2 years). Therefore, we cannot rule out that our population preferentially included the most MRSE tolerant patients with high levels of CPAP-usage.

The long-term design of our study is both a strength (that sets it apart from previous studies) and a limitation. Patients may have been treated with several masks and mask-types before inclusion and we cannot determine if the prevalence/severity of MRSEs were impacted by different mask sequences. Finally, our conclusions may not be applicable to short-term situations (< 3 months) or a younger/older patient population than that included in our study.

## Conclusions

In long-term CPAP-treated patients, patient-reported leaks are counter-intuitively not associated with device-reported leaks, and remain the most prevalent side effect. Additionally, MRSEs are associated with CPAP-non-adherence and RES. Considering the future care of millions of patients on a long-term basis, our study suggests a complementary role for MRSE-questionnaires and CPAP-reported data during telemedicine [34, 35].

## Supplementary Information

**Additional file 1.** Methods.

**Additional file 2.** Information (leaks and pressures) obtained by downloading device data according to manufacturer and device.

**Additional file 3.** Device reported leak data.

**Additional file 4.** Populations of patients depending on transformed manufacturer expressions of leaks.

**Additional file 5.** Mask characteristics (n = 1484).

**Additional file 6.** Explicative variables used during univariate analyses.

**Additional file 7.** Statistical analyses.

**Additional file 8.** Distribution frequency of the Epworth Sleepiness Scale (ESS) scores. The dashed line corresponds to cumulative frequency.

**Additional file 9.** Brand, series and mode of device used by patients.

**Additional file 10.** Univariate linear regression with patient-reported leak (0-10 VAS score) as the variable-of-interest.

**Additional file 11.** Multivariable linear regressions with patient-reported leak as the variable-of-interest (0-10 VAS score), summary of significant explanatory-variables.

**Additional file 12.** Association of mask related side-effects.

**Additional file 13.** Visual Analogue Scales for mask related side-effect scores (0-10 score) according to the presence/absence of a bed partner.

**Additional file 14.** Mask related side-effects categorized by previous (before 2013) or recent masks (since 2013) and subgroups of masks (nasal, oronasal and nasal pillows).

**Additional file 15.** Summary of the three most recent and largest cohort studies (>500 patients) reporting mask related side-effects in CPAP-treated patients.

## Data Availability

The datasets used and/or analyzed during the current study are available from the corresponding author on reasonable request.

## Abbreviations

AHI: Apnea–Hypopnea-Index
CPAP: Continuous positive airway pressure
ESS: Epworth Sleepiness Scale
MRSEs: Mask related side-effects
NM: Nasal mask
NPM: Nasal pillow mask
ONM: Oronasal mask
PCA: Principal component analysis
