# Supplementary material for: Mask side-effects in long-term CPAP-patients impact adherence and sleepiness: the InterfaceVent real-life study
Source: Respir Res. 2021 Jan 15;22:17. doi: 10.1186/s12931-021-01618-x (PMC7809735; doi:10.1186/s12931-021-01618-x)
Supplement: Supplementary file 1 — Additional file 1. Methods. [file 12931_2021_1618_MOESM1_ESM.docx]

**Title:**

Mask side-effects in long-term CPAP-patients impact adherence and sleepiness: the InterfaceVent real-life study.

**Authors:**

Marie-Caroline Rotty, BSc(Stat)^1,2^, Carey M. Suehs PhD^3,4^, Jean-Pierre Mallet MD^2,3^, Christian Martinez^2^, Jean-Christian Borel PhD^5^, Claudio Rabec MD^6^, Fanny Bertelli BSc(Stat)^1,2^, Arnaud Bourdin MD, PhD^2,3,7^, Nicolas Molinari PhD^1,3^, and Dany Jaffuel MD, PhD^2,3,7,8^.

**Affiliations:**

^1^ IMAG, CNRS, Montpellier University, Montpellier University Hospital, Montpellier, France.

^2^ Apard groupe Adène, Montpellier, France.

^3^ Department of Respiratory Diseases, Montpellier University Hospital, Arnaud de Villeneuve Hospital, Montpellier, France.

^4^ Department of Medical Information, Montpellier University Hospital, Montpellier, France.

^5^Grenoble Alps University, Inserm U1042, HP2 (Hypoxia PhysioPathology) Laboratory, Centre Hospitalier Universitaire Grenoble Alpes, Grenoble, France.

^6^Pulmonary Department and Respiratory Critical Care Unit, University Hospital Dijon, Dijon, France.

^7^ PhyMedExp (INSERM U 1046, CNRS UMR9214), Montpellier University, Montpellier, France.

^8^Pulmonary Disorders and Respiratory Sleep Disorders Unit, Polyclinic Saint-Privat, Boujan sur Libron, France.

**Corresponding author:**

Jaffuel Dany, Department of Respiratory Diseases, CHRU Montpellier, 371, Avenue Doyen Giraud, 34295 Montpellier Cedex 5, France. E-mail: [dany.jaffuel@wanadoo.fr](mailto:dany.jaffuel@wanadoo.fr)

Tel: +33661533104 ; Fax : +33467316484

**Additional File 1. Methods**

*Study design and study population, exclusion criteria*

Patient inclusions were performed from February 7, 2017 to April 1, 2019. For each patient included, only one data collection was performed. Demographic data, MRSE visual analogue scales, Epworth-Sleepiness-Scales and EQ-5D-3L questionnaires were collected only at the date of inclusion in the study. For CPAP-data, data were collected only at the time of inclusion using CPAP-software for the last 6 months for patients with a CPAP-duration treatment over 6 months, and for the last 3 months for patients with CPAP-duration duration from 3 months to 6 months. The CPAP-usage included in the interaction effects corresponds to the CPAP-usage collected at the time of inclusion (using CPAP-software for the previous 6 months for patients with a CPAP-duration treatment over 6 months, and for the last 3 months for patients with CPAP-duration from 3 to 6 months).”

Exclusion criteria were planned pregnancy, breastfeeding, inability to understand study nature/aims or to communicate with the investigator, simultaneous participation in another trial with an exclusion clause, non-affiliation with the French social security system, any kind of guardianship or judicial protection, use of an intra-oral or more-than-one mask.

*Epworth-Sleepiness-Scale (ESS)*

The ESS is the most widely used tool for assessing subjective sleepiness in research and clinical practice [1–3] The ESS was administered during the scheduled annual home visit by the technician for CPAP treatment control according to French Social Security rules. RES was defined as an ESS score of ≥11.

*EQ-5D-3L questionnaire*

The 3-L version of the EQ-5D (EQ-5D-3L) questionnaire was released in 1990 [4]. It comprises the following 5 dimensions: mobility, self-care, usual activities, pain/discomfort and anxiety/depression. Each dimension has 3 levels: no problems, some problems and extreme problems. The EQ VAS records the respondent’s self-rated health on a vertical, visual analogue scale where the endpoints are labelled ‘best imaginable health state’ and ‘worst imaginable health state’. This information can be used as a quantitative measure of health outcome as judged by the individual respondents.

*CPAP-reported leaks (CPAP_leaks_)*

Four manufacturer’s leak data were recorded. These data were used for descriptive statistics in Table 1 (unintentional leaks (l/min), unintentional large leaks (%), global leaks (l/min), global large leaks (%)). To render these data expressed in “l/min” or “%” as comparable as possible, a new pooled categorical variable named “device reported leaks” was created and used for multivariable regression analyses. The algorithm and rules to create this variable were as follows:

- First, patients must be included only one time in the final pooled variable. Some patients have two leak datasets/recordings. For example, the information contained in global large leaks expressed as “%” (n=137) is a subset of the information described by global leaks “l/min” (n=148), and is thus redundant. Consequently, only global leaks “l/min” has been kept for the next step. Similarly, for patients with both unintentional large leaks expressed in “%” (n=502) and also in “l/min” (n=898), only the latter was kept for the next step.

- Second, for each remaining variable (unintentional leaks, unintentional large leaks and global leaks), the corresponding leak data includes minimum and maximum values. The minimum was reclassified as “0” and the maximum was reclassified as “100”. The other values were interpolated between 0 and 100 accordingly. Additional file 3 and 4 depict the populations corresponding to these three transformed machine-provided leak variables.

-Third, these three leak variables were pooled into a single variable (ranging from 0 to 100) named “device reported leaks”. This new variable was used for multivariable regression analyses.

*Home Care Provider Policies*

Before reporting the APARD program and patient care policy details, it is important to keep in mind that i) policies have changed over time because of changes in the French Security Social reimbursement rules; ii) policies have changed over time because of increasingly innovative technologies used for patient care.

In detail, in the beginning of the 2000’s, four physical visits per year at the patient home by the home care provider were required by the FSS in order to comply with the FSS rules. The first physical visit following the CPAP-initiation was performed between the third and fourth weeks.

For the year 2017 and the following, the APARD program and policies included: a phone call two days to seven days after the CPAP-initiation, a physical visit before the third week, a physical visit at the fourth month, a physical visit once per year, a phone call every six months in the absence of physical visit. The telemedicine program included at least CPAP-adherence measures and phone calls in the case of decreasing CPAP-adherence to below 112 hours/28 days. As concerns innovations, the 32 technicians have an unrestricted access to masks (34 different masks) and market-available CPAP devices (5 manufacturers). In accordance to the 2010 French national recommendations [5], it was suggested to use a nasal mask as the first intention mask in newly CPAP-treated patients. To date there is no systematic policy for a mask-type change in the case of MRSEs occurrence. For example, for patient reported leaks associated with mouth leaks symptoms, the technician can propose a heated humidifier, heated breathing tube, chinstrap, or a switch to an oronasal type. The therapeutic choice results from the combination of the technician’s own experience and patient preferences. Nasal corticosteroid spray, CPAP pressure modification or comfort mode (CFlex, EPR…) require referent physician prescription. Before the InterfaceVent study, no systematic MRSE questionnaire was used, only an Epworth Scale was performed.

*References*

1. Johns MW. A new method for measuring daytime sleepiness: the Epworth sleepiness scale. Sleep. 1991;14:540–5.

2. Pépin J-L, Viot-Blanc V, Escourrou P, Racineux J-L, Sapene M, Lévy P, et al. Prevalence of residual excessive sleepiness in CPAP-treated sleep apnoea patients: the French multicentre study. Eur Respir J. 2009;33:1062–7.

3. Koutsourelakis I, Perraki E, Economou NT, Dimitrokalli P, Vagiakis E, Roussos C, et al. Predictors of residual sleepiness in adequately treated obstructive sleep apnoea patients. Eur Respir J. 2009;34:687–93.

4. EuroQol Group. EuroQol--a new facility for the measurement of health-related quality of life. Health Policy Amst Neth. 1990;16:199–208.

5. Société de Pneumologie de Langue Française, Société Française d’Anesthésie Réanimation, Société Française de Cardiologie, Société Française de Médecine du Travail, Société Française d’ORL, Société de Physiologie, et al. [Recommendations for clinical practice. Obstructive sleep apnea hypopnea syndrome in adults]. Rev Mal Respir. 2010;27:806–33.
