# Supplementary material for: Mask side-effects in long-term CPAP-patients impact adherence and sleepiness: the InterfaceVent real-life study
Source: Respir Res. 2021 Jan 15;22:17. doi: 10.1186/s12931-021-01618-x (PMC7809735; doi:10.1186/s12931-021-01618-x)
Supplement: Supplementary file 2 — Additional file 2. Information (leaks and pressures) obtained by downloading device data according to manufacturer and device. [file 12931_2021_1618_MOESM2_ESM.docx]

**Title:**

Mask side-effects in long-term CPAP-patients impact adherence and sleepiness: the InterfaceVent real-life study.

**Authors:**

Marie-Caroline Rotty, BSc(Stat)^1,2^, Carey M. Suehs PhD^3,4^, Jean-Pierre Mallet MD^2,3^, Christian Martinez^2^, Jean-Christian Borel PhD^5^, Claudio Rabec MD^6^, Fanny Bertelli BSc(Stat)^1,2^, Arnaud Bourdin MD, PhD^2,3,7^, Nicolas Molinari PhD^1,3^, and Dany Jaffuel MD, PhD^2,3,7,8^.

**Affiliations:**

^1^ IMAG, CNRS, Montpellier University, Montpellier University Hospital, Montpellier, France.

^2^ Apard groupe Adène, Montpellier, France.

^3^ Department of Respiratory Diseases, Montpellier University Hospital, Arnaud de Villeneuve Hospital, Montpellier, France.

^4^ Department of Medical Information, Montpellier University Hospital, Montpellier, France.

^5^Grenoble Alps University, Inserm U1042, HP2 (Hypoxia PhysioPathology) Laboratory, Centre Hospitalier Universitaire Grenoble Alpes, Grenoble, France.

^6^Pulmonary Department and Respiratory Critical Care Unit, University Hospital Dijon, Dijon, France.

^7^ PhyMedExp (INSERM U 1046, CNRS UMR9214), Montpellier University, Montpellier, France.

^8^Pulmonary Disorders and Respiratory Sleep Disorders Unit, Polyclinic Saint-Privat, Boujan sur Libron, France.

**Corresponding author:**

Jaffuel Dany, Department of Respiratory Diseases, CHRU Montpellier, 371, Avenue Doyen Giraud, 34295 Montpellier Cedex 5, France. E-mail: [dany.jaffuel@wanadoo.fr](mailto:dany.jaffuel@wanadoo.fr)

Tel: +33661533104 ; Fax : +33467316484

**Additional file 2. Information (leaks and pressures) obtained by downloading device data according to manufacturer and device.**

| **Information (leaks and pressures) obtained by downloading device data according to manufacturer and device (n: number of patients)** | | | | | | |
| --- | --- | --- | --- | --- | --- | --- |
| **Manufacturer and device** | **Fisher & Paykel**  (Icon ™, n=8)) | **Lowenstein Weinmann** | | **Philips**  (Remstar™, n=245 Dreamstation™, n=115) | **ResMed**  (S8™, n=51, S9™, n=381, S10™, n=335) | **Sefam**  (DreamStar™, n=130) |
|  |  | Prisma™, n=134 | Somnosmart 2™, n=95 |  |  |  |
| **Software Version** | F&P InfoSmart™  1.3.1 | PrismaTS™  4.1.0.37 | Somno support™  V 1.19 or V3.15 | EncorePro™  V2.20.6.0 or V2.21.4.1 | ResScan™  V5.8.0.9556 | DreamStar™  V5.1.0 |
| **Leaks** | Global leaks  (mean, L/min)  Global large leaks  (mean, %)  90^th^ percentile leaks (mean, L/min) | Unintentional leaks  (median, L/min)  Unintentional large leaks  (median, %)  95^th^ percentile leaks  (median, L/min) | Unintentional large leaks  (mean, %) | Unintentional  large leaks (mean, %)  Global leaks  (mean, L/min)  90^th^ percentile leaks  (mean, L/min) | Unintentional leaks  (median, L/min)  95^th^ percentile leaks  (median, L/min) | Global leaks  (mean, L/min)  Global large leaks  (mean, %) |
| **Pressure (cmH_2_O)** | Mean pressure  90^th^ percentile  mean pressure | Median pressure  90^th^ percentile  median pressure | Mean pressure  90^th^ and 95^th^ percentile  mean pressure | Mean pressure  90^th^ percentile  mean pressure | Median pressure  95^th^ percentile  median pressure | Mean pressure  90^th^ percentile  mean pressure |
