# Supplementary material for: Mask side-effects in long-term CPAP-patients impact adherence and sleepiness: the InterfaceVent real-life study
Source: Respir Res. 2021 Jan 15;22:17. doi: 10.1186/s12931-021-01618-x (PMC7809735; doi:10.1186/s12931-021-01618-x)
Supplement: Supplementary file 3 — Additional file 3. Device reported leak data. [file 12931_2021_1618_MOESM3_ESM.docx]

**Title:**

Mask side-effects in long-term CPAP-patients impact adherence and sleepiness: the InterfaceVent real-life study.

**Authors:**

Marie-Caroline Rotty, BSc(Stat)^1,2^, Carey M. Suehs PhD^3,4^, Jean-Pierre Mallet MD^2,3^, Christian Martinez^2^, Jean-Christian Borel PhD^5^, Claudio Rabec MD^6^, Fanny Bertelli BSc(Stat)^1,2^, Arnaud Bourdin MD, PhD^2,3,7^, Nicolas Molinari PhD^1,3^, and Dany Jaffuel MD, PhD^2,3,7,8^.

**Affiliations:**

^1^ IMAG, CNRS, Montpellier University, Montpellier University Hospital, Montpellier, France.

^2^ Apard groupe Adène, Montpellier, France.

^3^ Department of Respiratory Diseases, Montpellier University Hospital, Arnaud de Villeneuve Hospital, Montpellier, France.

^4^ Department of Medical Information, Montpellier University Hospital, Montpellier, France.

^5^Grenoble Alps University, Inserm U1042, HP2 (Hypoxia PhysioPathology) Laboratory, Centre Hospitalier Universitaire Grenoble Alpes, Grenoble, France.

^6^Pulmonary Department and Respiratory Critical Care Unit, University Hospital Dijon, Dijon, France.

^7^ PhyMedExp (INSERM U 1046, CNRS UMR9214), Montpellier University, Montpellier, France.

^8^Pulmonary Disorders and Respiratory Sleep Disorders Unit, Polyclinic Saint-Privat, Boujan sur Libron, France.

**Corresponding author:**

Jaffuel Dany, Department of Respiratory Diseases, CHRU Montpellier, 371, Avenue Doyen Giraud, 34295 Montpellier Cedex 5, France. E-mail: [dany.jaffuel@wanadoo.fr](mailto:dany.jaffuel@wanadoo.fr)

Tel: +33661533104 ; Fax : +33467316484

**Additional file 3. Device reported leak data**

| **Device reported leak data** | | | | | |
| --- | --- | --- | --- | --- | --- |
|  | Whole population  (n= 1484) | Nasal  (n= 807) | Oronasal  (n= 422) | Nasal pillows  (n= 255) | p-value |
| **Initial device-generated leak variables** |  |  |  |  |  |
| Unintentional Leaks (l/min), n= 898 (max= 91) | 2.5 [0; 7.5] | 2.5 [0; 8.4]^a^ | 1.2 [0; 6]^b^ | 1.5 [0; 7]^ab^ | 0.0002 |
| Unintentional Large Leaks (%), n= 502 (max= 100) | 0.1 [0; 1.1] | 0 [0; 0.9]^a^ | 0.2 [0; 3.0]^b^ | 0.1 [0; 0.9]^ac^ | 0.02 |
| Global Leaks (l/min), n= 148 (max= 70) | 33 [27; 41] | 31.5 [26; 37]^a^ | 37 (32; 49]^b^ | 32 [26; 41]^ab^ | 0.014 |
| Global Large Leaks (%), n= 137 | 0.9 [0.1; 5.1] | 0.7 [0.1; 3.1]^a^ | 2.7 [0.2; 6.7]^b^ | 0.9 [0.2; 5]^ac^ | 0.11 |
| **Transformed device-generated leak variables** |  |  |  |  |  |
| Unintentional Leaks (0-100 score), n= 898 | 2.7 [0; 8.2] | 2.7 [0; 9.2]^a^ | 1.3 [0; 6.6]^b^ | 1.6 [0; 7.7]^ab^ | 0.0002 |
| Unintentional Large Leaks (0-100 score), n= 403 | 0.1 [0; 0.9] | 0.1 [0; 0.6]^a^ | 0.1 [0; 2.5]^b^ | 0.1 [0; 0.7]^bc^ | 0.08 |
| Global Leaks (0-100 score), n= 148 | 47.1 [38.6; 58.6] | 45 [37.1; 52.9]^a^ | 52.9 [45.7; 70]^b^ | 45.7 [37.1; 58.6]^ab^ | 0.014 |
| **Pooled device-generated leak variables** |  |  |  |  |  |
| Device reported leaks (0-100 score), n=1449 | 1.9 [0; 9.2] | 2.6 [0; 9.2]^a^ | 1.3 [0; 8.2]^b^ | 1.3 [0; 8.2]^ab^ | 0.0357 |
| a,b,c: within a given line, subgroups with different letters are significantly different (p<0.05) according to post-hoc comparisons. | | | | | |
