# Supplementary material for: Mask side-effects in long-term CPAP-patients impact adherence and sleepiness: the InterfaceVent real-life study
Source: Respir Res. 2021 Jan 15;22:17. doi: 10.1186/s12931-021-01618-x (PMC7809735; doi:10.1186/s12931-021-01618-x)
Supplement: Supplementary file 5 — Additional file 5. Mask characteristics (n = 1484). [file 12931_2021_1618_MOESM5_ESM.docx]

**Title:**

Mask side-effects in long-term CPAP-patients impact adherence and sleepiness: the InterfaceVent real-life study.

**Authors:**

Marie-Caroline Rotty, BSc(Stat)^1,2^, Carey M. Suehs PhD^3,4^, Jean-Pierre Mallet MD^2,3^, Christian Martinez^2^, Jean-Christian Borel PhD^5^, Claudio Rabec MD^6^, Fanny Bertelli BSc(Stat)^1,2^, Arnaud Bourdin MD, PhD^2,3,7^, Nicolas Molinari PhD^1,3^, and Dany Jaffuel MD, PhD^2,3,7,8^.

**Affiliations:**

^1^ IMAG, CNRS, Montpellier University, Montpellier University Hospital, Montpellier, France.

^2^ Apard groupe Adène, Montpellier, France.

^3^ Department of Respiratory Diseases, Montpellier University Hospital, Arnaud de Villeneuve Hospital, Montpellier, France.

^4^ Department of Medical Information, Montpellier University Hospital, Montpellier, France.

^5^Grenoble Alps University, Inserm U1042, HP2 (Hypoxia PhysioPathology) Laboratory, Centre Hospitalier Universitaire Grenoble Alpes, Grenoble, France.

^6^Pulmonary Department and Respiratory Critical Care Unit, University Hospital Dijon, Dijon, France.

^7^ PhyMedExp (INSERM U 1046, CNRS UMR9214), Montpellier University, Montpellier, France.

^8^Pulmonary Disorders and Respiratory Sleep Disorders Unit, Polyclinic Saint-Privat, Boujan sur Libron, France.

**Corresponding author:**

Jaffuel Dany, Department of Respiratory Diseases, CHRU Montpellier, 371, Avenue Doyen Giraud, 34295 Montpellier Cedex 5, France. E-mail: [dany.jaffuel@wanadoo.fr](mailto:dany.jaffuel@wanadoo.fr)

Tel: +33661533104 ; Fax : +33467316484

**Additional file 5. Mask characteristics (n=1484)**

| **Mask characteristics (n=1484)** | | | | |
| --- | --- | --- | --- | --- |
| **Manufacturer** | **Model** | **Type of mask** | **Release date** | **n** |
| ResMed | Mirage Fx | Nasal | 2010 | 284 |
|  | Ultra mirage | Nasal | 2000 | 3 |
|  | Micro | Nasal | 2007 | 5 |
|  | Airfit N10 | Nasal | 2014 | 11 |
|  | Airfit F10 | Oronasal | 2014 | 66 |
|  | Airfit F20 | Oronasal | 2016 | 32 |
|  | Swift II | Nasal pillows | 2008 | 1 |
|  | Swift Fx | Nasal pillows | 2009 | 124 |
|  | Airfit P10 | Nasal pillows | 2013 | 6 |
|  | Quattro | Oronasal | 2007 | 144 |
|  | Quattro Fx ventil | Oronasal | 2010 | 3 |
| Fisher & Paykel | 405 | Nasal | 2003 | 5 |
|  | Hc 406 | Nasal | 2005 | 2 |
|  | Eson 1 | Nasal | 2012 | 160 |
|  | Eson 2 | Nasal | 2015 | 213 |
|  | Simplus | Oronasal | 2013 | 151 |
|  | Opus | Nasal pillows | 2007 | 28 |
|  | Pilairo | Nasal pillows | 2013 | 4 |
|  | Hc 407 | Nasal | 2005 | 54 |
|  | Forma | Oronasal | 2009 | 5 |
| L3 medical | Aura | Nasal | 2014 | 1 |
|  | Iq blue | Nasal | 2012 | 1 |
|  | Sleepweaver advance | Nasal | 2011 | 1 |
| Löwenstein Medical Technology | Joyce Silkgel | Nasal | 2010 | 38 |
|  | Joyce easy | Nasal | 2012 | 2 |
|  | Joyce one | Nasal | 2013 | 12 |
|  | Joyce one full face | Oronasal | 2014 | 19 |
| Philips | Conforgel | Nasal | 2003 | 1 |
|  | Amara view | Oronasal | 2015 | 2 |
|  | Nuance pro | Nasal pillows | 2014 | 88 |
|  | Dreamwear | Nasal pillows | 2014 | 4 |
|  | Easylife | Nasal | 2010 | 2 |
|  | Prolife | Nasal | 2000 | 7 |
| Sefam | Breeze | Nasal pillows | 2014 | 1 |
| Apard | Custom Made | Nasal | NA | 2 |
|  | Custom Made | Nasal pillows | NA | 1 |
|  | Custom Made | Oronasal | NA | 1 |

n= number; NA: Not Applicable
