# Supplementary material for: Mask side-effects in long-term CPAP-patients impact adherence and sleepiness: the InterfaceVent real-life study
Source: Respir Res. 2021 Jan 15;22:17. doi: 10.1186/s12931-021-01618-x (PMC7809735; doi:10.1186/s12931-021-01618-x)
Supplement: Supplementary file 6 — Additional file 6. Explicative variables used during univariate analyses. [file 12931_2021_1618_MOESM6_ESM.docx]

**Title:**

Mask side-effects in long-term CPAP-patients impact adherence and sleepiness: the InterfaceVent real-life study.

**Authors:**

Marie-Caroline Rotty, BSc(Stat)^1,2^, Carey M. Suehs PhD^3,4^, Jean-Pierre Mallet MD^2,3^, Christian Martinez^2^, Jean-Christian Borel PhD^5^, Claudio Rabec MD^6^, Fanny Bertelli BSc(Stat)^1,2^, Arnaud Bourdin MD, PhD^2,3,7^, Nicolas Molinari PhD^1,3^, and Dany Jaffuel MD, PhD^2,3,7,8^.

**Affiliations:**

^1^ IMAG, CNRS, Montpellier University, Montpellier University Hospital, Montpellier, France.

^2^ Apard groupe Adène, Montpellier, France.

^3^ Department of Respiratory Diseases, Montpellier University Hospital, Arnaud de Villeneuve Hospital, Montpellier, France.

^4^ Department of Medical Information, Montpellier University Hospital, Montpellier, France.

^5^Grenoble Alps University, Inserm U1042, HP2 (Hypoxia PhysioPathology) Laboratory, Centre Hospitalier Universitaire Grenoble Alpes, Grenoble, France.

^6^Pulmonary Department and Respiratory Critical Care Unit, University Hospital Dijon, Dijon, France.

^7^ PhyMedExp (INSERM U 1046, CNRS UMR9214), Montpellier University, Montpellier, France.

^8^Pulmonary Disorders and Respiratory Sleep Disorders Unit, Polyclinic Saint-Privat, Boujan sur Libron, France.

**Corresponding author:**

Jaffuel Dany, Department of Respiratory Diseases, CHRU Montpellier, 371, Avenue Doyen Giraud, 34295 Montpellier Cedex 5, France. E-mail: [dany.jaffuel@wanadoo.fr](mailto:dany.jaffuel@wanadoo.fr)

Tel: +33661533104 ; Fax : +33467316484

| **Additional file 6.**  **Explicative variables used during univariate analyses** | |  | |
| --- | --- | --- | --- |
| **Demographics** |  |  |  |
| Age (yrs) | | |  |
| Gender, female | | |  |
| BMI (kg/m²) | | |  |
| Diagnostic AHI (events/h) | | |  |
| Active smokers | | |  |
| Beard | | |  |
| Mustache | | |  |
| No mustache no beard | | |  |
| Active workers | | |  |
| Presence of partner | | |  |
| **Epworth Scale** | | |  |
| ESS (0-24 scores) | | |  |
| **EQ-5D-3L** | | |  |
| Problems with mobility | | |  |
| Problems with self-care | | |  |
| Problems with usual activities | | |  |
| Problems of pain/discomfort | | |  |
| Problems of anxiety/depression | | |  |
| EQ-5D-3L health VAS (0-100 score) | | |  |
| **Device** | | |  |
| CPAP-usage (h/day) | | |  |
| Current AHI_flow_ (events/h) | | |  |
| Treatment duration (yrs) | | |  |
| Fixed pressure | | |  |
| Mean Pressure (cmH_2_O) | | |  |
| 90^th^/95^th^ pressure (cmH_2_O) | | |  |
| Comfort mode | | |  |
| Heated humidifier | | |  |
| Heated breathing tube | | |  |
| **Mask** | | |  |
| Nasal Mask | | |  |
| Oronasal Mask | | |  |
| Nasal Pillows Mask | | |  |
| Availability of the mask since 2013 (%) | | |  |
| Device reported leaks (0-100 score) | | |  |
| Device reported leaks (median of the 95^th^ percentile of unintentional leaks (l/min)) | | |  |
| Chin strap | | |  |
| **Side effects** | | |  |
| Dry mouth (0-10 VAS score) | | |  |
| Partner disturbing leaks (0-10 VAS score) | | |  |
| Patient reported leaks (0-10 VAS score) | | |  |
| Red eyes (0-10 VAS score) | | |  |
| Itchy eyes (0-10 VAS score) | | |  |
| Noisy mask (0-10 VAS score) | | |  |
| Dry nose (0-10 VAS score) | | |  |
| Stuffed nose (0-10 VAS score) | | |  |
| Runny nose (0-10 VAS score) | | |  |
| Heavy mask (0-10 VAS score) | | |  |
| Mask pain (0-10 VAS score) | | |  |
| Mask injury (0-10 VAS score) | | |  |
| Harness pain (0-10 VAS score) | | |  |
| Harness injury (0-10 VAS score) | | |  |
| Nose bleeding | | |  |
| Aerophagia | | |  |
| Number* (0/14) | | |  |
