# Supplementary material for: Mask side-effects in long-term CPAP-patients impact adherence and sleepiness: the InterfaceVent real-life study
Source: Respir Res. 2021 Jan 15;22:17. doi: 10.1186/s12931-021-01618-x (PMC7809735; doi:10.1186/s12931-021-01618-x)
Supplement: Supplementary file 7 — Additional file 7. Statistical analyses. [file 12931_2021_1618_MOESM7_ESM.docx]

**Title:**

Mask side-effects in long-term CPAP-patients impact adherence and sleepiness: the InterfaceVent real-life study.

**Authors:**

Marie-Caroline Rotty, BSc(Stat)^1,2^, Carey M. Suehs PhD^3,4^, Jean-Pierre Mallet MD^2,3^, Christian Martinez^2^, Jean-Christian Borel PhD^5^, Claudio Rabec MD^6^, Fanny Bertelli BSc(Stat)^1,2^, Arnaud Bourdin MD, PhD^2,3,7^, Nicolas Molinari PhD^1,3^, and Dany Jaffuel MD, PhD^2,3,7,8^.

**Affiliations:**

^1^ IMAG, CNRS, Montpellier University, Montpellier University Hospital, Montpellier, France.

^2^ Apard groupe Adène, Montpellier, France.

^3^ Department of Respiratory Diseases, Montpellier University Hospital, Arnaud de Villeneuve Hospital, Montpellier, France.

^4^ Department of Medical Information, Montpellier University Hospital, Montpellier, France.

^5^Grenoble Alps University, Inserm U1042, HP2 (Hypoxia PhysioPathology) Laboratory, Centre Hospitalier Universitaire Grenoble Alpes, Grenoble, France.

^6^Pulmonary Department and Respiratory Critical Care Unit, University Hospital Dijon, Dijon, France.

^7^ PhyMedExp (INSERM U 1046, CNRS UMR9214), Montpellier University, Montpellier, France.

^8^Pulmonary Disorders and Respiratory Sleep Disorders Unit, Polyclinic Saint-Privat, Boujan sur Libron, France.

**Corresponding author:**

Jaffuel Dany, Department of Respiratory Diseases, CHRU Montpellier, 371, Avenue Doyen Giraud, 34295 Montpellier Cedex 5, France. E-mail: [dany.jaffuel@wanadoo.fr](mailto:dany.jaffuel@wanadoo.fr)

Tel: +33661533104 ; Fax : +33467316484

**Additional file 7. Statistical analyses**

**PCA analysis**

To visualize correlations between MRSEs for a given mask type, a principal component analysis was performed for nasal, oronasal and nasal pillows mask. PCA was implemented using proc princomp (factor analysis and data mining with SAS, <https://documentation.sas.com/?docsetId=statug&docsetTarget=statug_princomp_syntax01.htm&docsetVersion=15.1&locale=en>). PCA extracts a first factor representing as much of the variation in the data as possible (Axis 1), and further axes that are subsequently orthogonal to the previous.

**Multivariable models**

Depending on the variables-of-interest (CPAP-non-adherence, or Residual Excessive Sleepiness, or patient-reported leaks), different multivariable regression techniques were applied.

For model 1, explanatory-variables (exhaustively listed in Additional file 6) with a p-value <0.15 at the univariate level were fed into multivariable analyses using stepwise selection. A backward elimination was then applied and only explanatory-variables with a p value <0.05 at the multivariable level remain in the definitive models.

For model 2, the same proceeding as model 1 was applied and the “mask-type” explanatory variable was forced (with “nasal” as the reference mask type).

For model 3, the same procedure as model 2 was applied, but the interaction terms between CPAP-usage and significant MRSEs from model 2 were added and a backward elimination applied excepted for the “mask-type” variable which remains forced.
