# Supplementary material for: Mask side-effects in long-term CPAP-patients impact adherence and sleepiness: the InterfaceVent real-life study
Source: Respir Res. 2021 Jan 15;22:17. doi: 10.1186/s12931-021-01618-x (PMC7809735; doi:10.1186/s12931-021-01618-x)

**Title:**

Mask side-effects in long-term CPAP-patients impact adherence and sleepiness: the InterfaceVent real-life study.

**Authors:**

Marie-Caroline Rotty, BSc(Stat)^1,2^, Carey M. Suehs PhD^3,4^, Jean-Pierre Mallet MD^2,3^, Christian Martinez^2^, Jean-Christian Borel PhD^5^, Claudio Rabec MD^6^, Fanny Bertelli BSc(Stat)^1,2^, Arnaud Bourdin MD, PhD^2,3,7^, Nicolas Molinari PhD^1,3^, and Dany Jaffuel MD, PhD^2,3,7,8^.

**Affiliations:**

^1^ IMAG, CNRS, Montpellier University, Montpellier University Hospital, Montpellier, France.

^2^ Apard groupe Adène, Montpellier, France.

^3^ Department of Respiratory Diseases, Montpellier University Hospital, Arnaud de Villeneuve Hospital, Montpellier, France.

^4^ Department of Medical Information, Montpellier University Hospital, Montpellier, France.

^5^Grenoble Alps University, Inserm U1042, HP2 (Hypoxia PhysioPathology) Laboratory, Centre Hospitalier Universitaire Grenoble Alpes, Grenoble, France.

^6^Pulmonary Department and Respiratory Critical Care Unit, University Hospital Dijon, Dijon, France.

^7^ PhyMedExp (INSERM U 1046, CNRS UMR9214), Montpellier University, Montpellier, France.

^8^Pulmonary Disorders and Respiratory Sleep Disorders Unit, Polyclinic Saint-Privat, Boujan sur Libron, France.

**Corresponding author:**

Jaffuel Dany, Department of Respiratory Diseases, CHRU Montpellier, 371, Avenue Doyen Giraud, 34295 Montpellier Cedex 5, France. E-mail: [dany.jaffuel@wanadoo.fr](mailto:dany.jaffuel@wanadoo.fr)

Tel: +33661533104 ; Fax : +33467316484

**Additional file 8. Distribution frequency of the Epworth Sleepiness Scale (ESS) scores. The dashed line corresponds to cumulative frequency.**


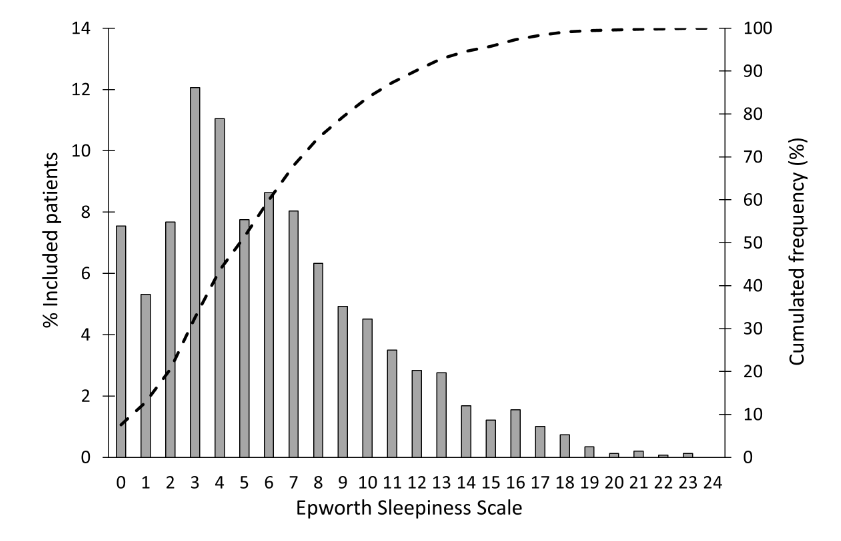

Supplement: Supplementary file 8 — Additional file 8. Distribution frequency of the Epworth Sleepiness Scale (ESS) scores. The dashed line corresponds to cumulative frequency. [file 12931_2021_1618_MOESM8_ESM.docx]
