# Supplementary material for: Mask side-effects in long-term CPAP-patients impact adherence and sleepiness: the InterfaceVent real-life study
Source: Respir Res. 2021 Jan 15;22:17. doi: 10.1186/s12931-021-01618-x (PMC7809735; doi:10.1186/s12931-021-01618-x)
Supplement: Supplementary file 9 — Additional file 9. Brand, series and mode of device used by patients. [file 12931_2021_1618_MOESM9_ESM.docx]

**Title:**

Mask side-effects in long-term CPAP-patients impact adherence and sleepiness: the InterfaceVent real-life study.

**Authors:**

Marie-Caroline Rotty, BSc(Stat)^1,2^, Carey M. Suehs PhD^3,4^, Jean-Pierre Mallet MD^2,3^, Christian Martinez^2^, Jean-Christian Borel PhD^5^, Claudio Rabec MD^6^, Fanny Bertelli BSc(Stat)^1,2^, Arnaud Bourdin MD, PhD^2,3,7^, Nicolas Molinari PhD^1,3^, and Dany Jaffuel MD, PhD^2,3,7,8^.

**Affiliations:**

^1^ IMAG, CNRS, Montpellier University, Montpellier University Hospital, Montpellier, France.

^2^ Apard groupe Adène, Montpellier, France.

^3^ Department of Respiratory Diseases, Montpellier University Hospital, Arnaud de Villeneuve Hospital, Montpellier, France.

^4^ Department of Medical Information, Montpellier University Hospital, Montpellier, France.

^5^Grenoble Alps University, Inserm U1042, HP2 (Hypoxia PhysioPathology) Laboratory, Centre Hospitalier Universitaire Grenoble Alpes, Grenoble, France.

^6^Pulmonary Department and Respiratory Critical Care Unit, University Hospital Dijon, Dijon, France.

^7^ PhyMedExp (INSERM U 1046, CNRS UMR9214), Montpellier University, Montpellier, France.

^8^Pulmonary Disorders and Respiratory Sleep Disorders Unit, Polyclinic Saint-Privat, Boujan sur Libron, France.

**Corresponding author:**

Jaffuel Dany, Department of Respiratory Diseases, CHRU Montpellier, 371, Avenue Doyen Giraud, 34295 Montpellier Cedex 5, France. E-mail: [dany.jaffuel@wanadoo.fr](mailto:dany.jaffuel@wanadoo.fr)

Tel: +33661533104 ; Fax : +33467316484

**Additional file 9. Brand, series and mode of device used by patients**

| **Brand, series and mode of device used by patients (n= 1484)** | | | |
| --- | --- | --- | --- |
| **Device** | **Number** | **Fixed Pressure**  **(n)** | **Auto** **Pressure**  **(n)** |
| **ResMed devices** | 767 | 121 | 646 |
| AIRSENSE 10 | 335 | 50 | 285 |
| AUTOSET S8 | 51 | 10 | 41 |
| AUTOSET S9 | 381 | 61 | 320 |
| **SEFAM devices** | 130 | 9 | 121 |
| DREAMSTAR AUTO | 130 | 9 | 121 |
| **PHILIPS devices** | 360 | 41 | 318 |
| REMSTAR AUTO | 245* | 34 | 210 |
| DREAMSTATION AUTO | 115 | 7 | 108 |
| **Fisher & Paykel devices** | 8 | 0 | 8 |
| ICON/ICON PLUS | 8 | 0 | 8 |
| **Löwenstein/Weinmann devices** | 219 | 20 | 199 |
| PRISMA20/PRISMASMART | 134 | 18 | 106 |
| SOMNOSMART 2 | 95 | 2 | 93 |
| *one device pressure mode unavailable, n= number. | | | |
