# Supplementary material for: Mask side-effects in long-term CPAP-patients impact adherence and sleepiness: the InterfaceVent real-life study
Source: Respir Res. 2021 Jan 15;22:17. doi: 10.1186/s12931-021-01618-x (PMC7809735; doi:10.1186/s12931-021-01618-x)
Supplement: Supplementary file 10 — Additional file 10. Univariate linear regression with patient-reported leak (0-10 VAS score) as the variable-of-interest. [file 12931_2021_1618_MOESM10_ESM.docx]

**Title:**

Mask side-effects in long-term CPAP-patients impact adherence and sleepiness: the InterfaceVent real-life study.

**Authors:**

Marie-Caroline Rotty, BSc(Stat)^1,2^, Carey M. Suehs PhD^3,4^, Jean-Pierre Mallet MD^2,3^, Christian Martinez^2^, Jean-Christian Borel PhD^5^, Claudio Rabec MD^6^, Fanny Bertelli BSc(Stat)^1,2^, Arnaud Bourdin MD, PhD^2,3,7^, Nicolas Molinari PhD^1,3^, and Dany Jaffuel MD, PhD^2,3,7,8^.

**Affiliations:**

^1^ IMAG, CNRS, Montpellier University, Montpellier University Hospital, Montpellier, France.

^2^ Apard groupe Adène, Montpellier, France.

^3^ Department of Respiratory Diseases, Montpellier University Hospital, Arnaud de Villeneuve Hospital, Montpellier, France.

^4^ Department of Medical Information, Montpellier University Hospital, Montpellier, France.

^5^Grenoble Alps University, Inserm U1042, HP2 (Hypoxia PhysioPathology) Laboratory, Centre Hospitalier Universitaire Grenoble Alpes, Grenoble, France.

^6^Pulmonary Department and Respiratory Critical Care Unit, University Hospital Dijon, Dijon, France.

^7^ PhyMedExp (INSERM U 1046, CNRS UMR9214), Montpellier University, Montpellier, France.

^8^Pulmonary Disorders and Respiratory Sleep Disorders Unit, Polyclinic Saint-Privat, Boujan sur Libron, France.

**Corresponding author:**

Jaffuel Dany, Department of Respiratory Diseases, CHRU Montpellier, 371, Avenue Doyen Giraud, 34295 Montpellier Cedex 5, France. E-mail: [dany.jaffuel@wanadoo.fr](mailto:dany.jaffuel@wanadoo.fr)

Tel: +33661533104 ; Fax : +33467316484

| **Additional file 10. Univariate linear regression with patient-reported leak (0-10 VAS score) as the variable-of-interest** | | |  |
| --- | --- | --- | --- |
|  | **Standardized**  **β coefficient** | **P value** | |
| **Demographics** |  |  | |
| Age (yrs) | -0.02 | 0.34 | |
| Gender, female | 0.01 | 0.82 | |
| BMI (kg/m²) | 0.03 | 0.89 | |
| Diagnostic AHI (events/h) | -0.02 | 0.38 | |
| Active smokers | 0.002 | 0.95 | |
| Beard | 0.005 | 0.87 | |
| Mustache | -0.004 | 0.90 | |
| Active workers | 0.02 | 0.52 | |
| Presence of partner | **0.04** | **0.14** | |
| **Epworth Scale** |  |  | |
| ESS (0-24 scores) | **0.22** | **<0.001** | |
| **EQ-5D-3L** |  |  | |
| Problems with mobility | 0.02 | 0.47 | |
| Problems with self-care | -0.009 | 0.75 | |
| Problems with usual activities | -0.02 | 0.53 | |
| Problems of pain/discomfort | 0.02 | 0.37 | |
| Problems of anxiety/depression | **0.05** | **0.040** | |
| EQ-5D-3L health VAS (0-100 score) | **-0.06** | **0.035** | |
| **Device** |  |  | |
| CPAP-usage (h/day) | **0.001** | 0.97 | |
| Current AHI_flow_ (events/h) | **0.009** | 0.72 | |
| Treatment duration (yrs) | 0.01 | 0.45 | |
| Fixed pressure | -0.02 | 0.44 | |
| Mean Pressure (cmH_2_O) | **0.07** | **0.009** | |
| 90^th^/95^th^ pressure (cmH_2_O) | **0.08** | **0.002** | |
| Comfort mode | -0.007 | 0.78 | |
| Heated humidifier | 0.04 | 0.16 | |
| Heated breathing tube | -0.007 | 0.79 | |
| **Mask** |  |  | |
| Nasal Mask | **Ref** | **<0.001** | |
| Oronasal Mask | **0.13** | **<0.001** | |
| Nasal Pillows Mask | **0.05** | **0.094** | |
| Availability of the mask since 2013 (%) | **0.07** | **0.010** | |
| Device reported leaks (0-100 score) | **-0.01** | 0.79 | |
| Device reported leaks (95^th^ percentile) | 0.01 | 0.71 | |
| Chin strap | 0.02 | 0.36 | |
| **Side effects** |  |  | |
| Dry mouth (0-10 VAS score) | **0.33** | **<0.001** | |
| Partner disturbing leaks (0-10 VAS score) | **0.43** | **<0.0001** | |
| Patient reported leaks (0-10 VAS score) | NA | NA | |
| Red eyes (0-10 VAS score) | **0.26** | **<0.001** | |
| Itchy eyes (0-10 VAS score) | **0.26** | **<0.001** | |
| Noisy mask (0-10 VAS score) | **0.48** | **<0.001** | |
| Dry nose (0-10 VAS score) | **0.26** | **<0.001** | |
| Stuffed nose (0-10 VAS score) | **0.26** | **<0.001** | |
| Runny nose (0-10 VAS score) | **0.16** | **<0.001** | |
| Heavy mask (0-10 VAS score) | **0.36** | **<0.001** | |
| Mask pain (0-10 VAS score) | **0.30** | **<0.001** | |
| Mask injury (0-10 VAS score) | **0.21** | **<0.001** | |
| Harness pain (0-10 VAS score) | **0.27** | **<0.001** | |
| Harness injury (0-10 VAS score) | **0.21** | **<0.001** | |
| Nose bleeding | **0.05** | **0.0647** | |
| Aerophagia | **0.12** | **<0.001** | |
| Number* (0/14) | 0.44 | <0.001 | |
