# Supplementary material for: Mask side-effects in long-term CPAP-patients impact adherence and sleepiness: the InterfaceVent real-life study
Source: Respir Res. 2021 Jan 15;22:17. doi: 10.1186/s12931-021-01618-x (PMC7809735; doi:10.1186/s12931-021-01618-x)
Supplement: Supplementary file 11 — Additional file 11. Multivariable linear regressions with patient-reported leak as the variable-of-interest (0-10 VAS score), summary of significant explanatory-variables. [file 12931_2021_1618_MOESM11_ESM.docx]

**Title:**

Mask side-effects in long-term CPAP-patients impact adherence and sleepiness: the InterfaceVent real-life study.

**Authors:**

Marie-Caroline Rotty, BSc(Stat)^1,2^, Carey M. Suehs PhD^3,4^, Jean-Pierre Mallet MD^2,3^, Christian Martinez^2^, Jean-Christian Borel PhD^5^, Claudio Rabec MD^6^, Fanny Bertelli BSc(Stat)^1,2^, Arnaud Bourdin MD, PhD^2,3,7^, Nicolas Molinari PhD^1,3^, and Dany Jaffuel MD, PhD^2,3,7,8^.

**Affiliations:**

^1^ IMAG, CNRS, Montpellier University, Montpellier University Hospital, Montpellier, France.

^2^ Apard groupe Adène, Montpellier, France.

^3^ Department of Respiratory Diseases, Montpellier University Hospital, Arnaud de Villeneuve Hospital, Montpellier, France.

^4^ Department of Medical Information, Montpellier University Hospital, Montpellier, France.

^5^Grenoble Alps University, Inserm U1042, HP2 (Hypoxia PhysioPathology) Laboratory, Centre Hospitalier Universitaire Grenoble Alpes, Grenoble, France.

^6^Pulmonary Department and Respiratory Critical Care Unit, University Hospital Dijon, Dijon, France.

^7^ PhyMedExp (INSERM U 1046, CNRS UMR9214), Montpellier University, Montpellier, France.

^8^Pulmonary Disorders and Respiratory Sleep Disorders Unit, Polyclinic Saint-Privat, Boujan sur Libron, France.

**Corresponding author:**

Jaffuel Dany, Department of Respiratory Diseases, CHRU Montpellier, 371, Avenue Doyen Giraud, 34295 Montpellier Cedex 5, France. E-mail: [dany.jaffuel@wanadoo.fr](mailto:dany.jaffuel@wanadoo.fr)

Tel: +33661533104 ; Fax : +33467316484

| **Additional file 11. Multivariable linear regressions with patient-reported leak as the variable-of-interest (0-10 VAS score), summary of significant explanatory-variables** | | | | | | |
| --- | --- | --- | --- | --- | --- | --- |
|  | **Model 1** | | **Model 2** | | **Model 3** | |
|  | Standardized  β coefficient | p-value | Standardized β coefficient | p-value | Standardized β coefficient | p-value |
| **Mask** |  |  |  |  |  |  |
| Oronasal Mask |  |  | 0.08 | 0.002 | 0.08 | 0.004 |
| Nasal Pillows Mask |  |  | 0.01 | 0.722 | 0.01 | 0.716 |
| Availability of the mask since 2013 | 0.06 | 0.012 |  |  |  |  |
| **Side-effects** |  |  |  |  |  |  |
| Dry mouth (0-10 VAS score) | 0.13 | <0.001 | 0.13 | <0.001 | 0.29 | <0.001 |
| Partner-disturbing leaks (0-10 VAS score) | 0.26 | <0.001 | 0.26 | <0.001 | 0.26 | <0.001 |
| Red eyes (0-10 VAS score) | 0.09 | <0.001 | 0.09 | <0.001 |  |  |
| Noisy mask (0-10 VAS score) | 0.33 | <0.001 | 0.28 | <0.001 | 0.28 | <0.001 |
| Stuffed Nose (0-10 VAS score) | 0.08 | 0.004 | 0.08 | 0.003 | 0.07 | 0.005 |
| Harness pain (0-10 VAS score) | 0.16 | <0.001 | 0.08 | 0.001 |  |  |
| **Interaction terms between CPAP-usage and MRSE** |  |  |  |  |  |  |
| CPAP-usage and dry mouth | NA | NA | NA | NA | -0.17 | 0.005 |
| CPAP-usage and red eyes | NA | NA | NA | NA | 0.11 | <0.001 |
| CPAP-usage and harness pain | NA | NA | NA | NA | 0.09 | <0.001 |
| R^2^ | 0.372  0.368 | | 0.374  0.369 | | 0.379  0.374 | |
| Adjusted R^2^ |  |  |  |  |  |  |
| CPAP: Continuous Positive Airway Pressure; NA: Not Applicable; VAS: Visual Analogue Scale.  Note that for model 1, explanatory-variables (exhaustively listed in Additional File 10) with a p-value <0.15 at the univariate level were fed into multivariable analyses using stepwise selection. A backward elimination was then applied and only explanatory-variables with a p value <0.05 at the multivariable level remain in the definitive model. For model 2, the same proceeding as model 1 was applied and the “mask-type” explanatory variable was forced (with “nasal” as the reference mask-type). For model 3, the same procedure as model 2 was applied, but the interaction terms between CPAP-usage and significant MRSEs from model 2 were added and a backward elimination applied excepted for the “mask-type” variable which remains forced. | | | | | | |
