# Supplementary material for: Mask side-effects in long-term CPAP-patients impact adherence and sleepiness: the InterfaceVent real-life study
Source: Respir Res. 2021 Jan 15;22:17. doi: 10.1186/s12931-021-01618-x (PMC7809735; doi:10.1186/s12931-021-01618-x)
Supplement: Supplementary file 12 — Additional file 12. Association of mask related side-effects. [file 12931_2021_1618_MOESM12_ESM.docx]

**Title:**

Mask side-effects in long-term CPAP-patients impact adherence and sleepiness: the InterfaceVent real-life study.

**Authors:**

Marie-Caroline Rotty, BSc(Stat)^1,2^, Carey M. Suehs PhD^3,4^, Jean-Pierre Mallet MD^2,3^, Christian Martinez^2^, Jean-Christian Borel PhD^5^, Claudio Rabec MD^6^, Fanny Bertelli BSc(Stat)^1,2^, Arnaud Bourdin MD, PhD^2,3,7^, Nicolas Molinari PhD^1,3^, and Dany Jaffuel MD, PhD^2,3,7,8^.

**Affiliations:**

^1^ IMAG, CNRS, Montpellier University, Montpellier University Hospital, Montpellier, France.

^2^ Apard groupe Adène, Montpellier, France.

^3^ Department of Respiratory Diseases, Montpellier University Hospital, Arnaud de Villeneuve Hospital, Montpellier, France.

^4^ Department of Medical Information, Montpellier University Hospital, Montpellier, France.

^5^Grenoble Alps University, Inserm U1042, HP2 (Hypoxia PhysioPathology) Laboratory, Centre Hospitalier Universitaire Grenoble Alpes, Grenoble, France.

^6^Pulmonary Department and Respiratory Critical Care Unit, University Hospital Dijon, Dijon, France.

^7^ PhyMedExp (INSERM U 1046, CNRS UMR9214), Montpellier University, Montpellier, France.

^8^Pulmonary Disorders and Respiratory Sleep Disorders Unit, Polyclinic Saint-Privat, Boujan sur Libron, France.

**Corresponding author:**

Jaffuel Dany, Department of Respiratory Diseases, CHRU Montpellier, 371, Avenue Doyen Giraud, 34295 Montpellier Cedex 5, France. E-mail: [dany.jaffuel@wanadoo.fr](mailto:dany.jaffuel@wanadoo.fr)

Tel: +33661533104 ; Fax : +33467316484

| **Additional file 12. Association of mask related side-effects** | | | |
| --- | --- | --- | --- |
| **Number of side-effects (VAS ≥ 1)** | **N (% of patients)** | **Number of side-effects (VAS ≥ 5)** | **N (% of patients)** |
| 0 | 94 (6.33) | 0 | 423 (28.5) |
| 1 | 107 (7.21) | 1 | 235 (15.8) |
| 2 | 127 (8.56) | 2 | 211 (14.2) |
| 3 | 155 (10.4) | 3 | 183 (12.3) |
| 4 | 168 (11.3) | 4 | 144 (9.70) |
| 5 | 145 (9.77) | 5 | 99 (6.67) |
| 6 | 123 (8.29) | 6 | 80 (5.39) |
| 7 | 97 (6.54) | 7 | 46 (3.10) |
| 8 | 90 (6.06) | 8 | 26 (1.75) |
| 9 | 57 (3.84) | 9 | 22 (1.48) |
| 10 | 62 (4.18) | 10 | 7 (0.47) |
| 11 | 49 (3.30) | 11 | 2 (0.13) |
| 12 | 24 (1.62) | 12 | 4 (0.27) |
| 13 | 58 (3.91) | 13 | 2 (0.13) |
| 14 | 128 (8.63) | 14 | 0 (0) |
| N= number of patient responding; VAS: Visual Analogue Scale. | | | |
