# Supplementary material for: Mask side-effects in long-term CPAP-patients impact adherence and sleepiness: the InterfaceVent real-life study
Source: Respir Res. 2021 Jan 15;22:17. doi: 10.1186/s12931-021-01618-x (PMC7809735; doi:10.1186/s12931-021-01618-x)
Supplement: Supplementary file 15 — Additional file 15. Summary of the three most recent and largest cohort studies (>500 patients) reporting mask related side-effects in CPAP-treated patients. [file 12931_2021_1618_MOESM15_ESM.docx]

| **Additional file 15.** **Summary of the three most recent and largest cohort studies (>500 patients) reporting mask related side-effects in CPAP-treated patients** | | | | |
| --- | --- | --- | --- | --- |
| **Study**  **/**  **Type of questionnaire** | **Number of patients**  **/**  **Duration of CPAP treatment**  **/**  **CPAP-usage (h/day)** | **Proportion of nasal, oronasal,**  **nasal pillow**  **masks** | **Mask related side-effects (three main MRSEs in % of patients)** | **Association of patient mask related side-effects with CPAP-non-adherence (<4h/day)**  **and/or residual excessive sleepiness (ESS≥11)** |
| Bachour et al. 2013 [1] VAS (0-100 score) | 730 patients  Mean 1.91±1.9 yrs  (range 0.06–16)  Mean 5.6±2.8 h/day | Nasal = 79 %  Oronasal = 9 %,  Nasal pillows = 9 % | Patient-reported leaks = 65%  CPAP mask pressure on the skin = 48%  Uncomfortable mask = 30% | No statistically significant association between the reported CPAP-non-adherence and the satisfaction rate with the mask. |
| Borel et al.2013 [2]  Binary questions | 2311 patients  Mean 0.38±0.3 yrs  Mean 5.39±1.92 h/day | Nasal = 62.4%  Oronasal = 26.2%  Nasal Pillows = 11.4% | Dry mouth = 25.3%  Nasal Congestion = 12.16%  Ocular irritation = 6.75% | Dry mouth and nasal congestion decrease the proportion of patients adherent to the CPAP (univariate analysis) |
| Rotty et al.  VAS (0-10 score) | 1484 patients  Median 4.4 years  [2.0; 9.7]  Median 6.8h/day  [5.5; 7.8] | Nasal = 54.4%  Oronasal = 28.4%  Nasal Pillows = 17.2% | Patient-reported leaks = 75.4%  Dry mouth = 70.6%  Noisy Mask = 57.5% | Higher VAS scores for dry mouth are associated with CPAP-non-adherence (multivariable analysis).  Higher VAS scores for patient-reported leaks, noisy mask, dry nose and harness pain are associated with an increased proportion of patients with RES (multivariable analysis). |

**Title:**

Mask side-effects in long-term CPAP-patients impact adherence and sleepiness: the InterfaceVent real-life study.

**Authors:**

Marie-Caroline Rotty, BSc(Stat)^1,2^, Carey M. Suehs PhD^3,4^, Jean-Pierre Mallet MD^2,3^, Christian Martinez^2^, Jean-Christian Borel PhD^5^, Claudio Rabec MD^6^, Fanny Bertelli BSc(Stat)^1,2^, Arnaud Bourdin MD, PhD^2,3,7^, Nicolas Molinari PhD^1,3^, and Dany Jaffuel MD, PhD^2,3,7,8^.

**Affiliations:**

^1^ IMAG, CNRS, Montpellier University, Montpellier University Hospital, Montpellier, France.

^2^ Apard groupe Adène, Montpellier, France.

^3^ Department of Respiratory Diseases, Montpellier University Hospital, Arnaud de Villeneuve Hospital, Montpellier, France.

^4^ Department of Medical Information, Montpellier University Hospital, Montpellier, France.

^5^Grenoble Alps University, Inserm U1042, HP2 (Hypoxia PhysioPathology) Laboratory, Centre Hospitalier Universitaire Grenoble Alpes, Grenoble, France.

^6^Pulmonary Department and Respiratory Critical Care Unit, University Hospital Dijon, Dijon, France.

^7^ PhyMedExp (INSERM U 1046, CNRS UMR9214), Montpellier University, Montpellier, France.

^8^Pulmonary Disorders and Respiratory Sleep Disorders Unit, Polyclinic Saint-Privat, Boujan sur Libron, France.

**Corresponding author:**

Jaffuel Dany, Department of Respiratory Diseases, CHRU Montpellier, 371, Avenue Doyen Giraud, 34295 Montpellier Cedex 5, France. E-mail: [dany.jaffuel@wanadoo.fr](mailto:dany.jaffuel@wanadoo.fr)

Tel: +33661533104 ; Fax : +33467316484

Values are expressed as they were reported in their original publication (e.g. mean or median). CPAP: Continuous Positive Airway Pressure; ESS: Epworth-Sleepiness-Scale; MRSE: Mask Related Side-Effect; RES: Residual Excessive Sleepiness (ESS score >10); VAS: Visual Analogue Scale.

References

1. Bachour A, Vitikainen P, Virkkula P, Maasilta P. CPAP interface: satisfaction and side effects. Sleep Breath. 2013;17:667–72.

2. Borel JC, Tamisier R, Dias-Domingos S, Sapene M, Martin F, Stach B, et al. Type of mask may impact on continuous positive airway pressure adherence in apneic patients. PloS One. 2013;8:e64382.
